# Patterns of Functional Diversity, Species Diversity, and Endemicity Driven by Elevation and Topographic Complexity in a Mediterranean Mountain Refuge

**DOI:** 10.1002/ece3.71354

**Published:** 2025-05-07

**Authors:** Candan Aykurt, Kürşad Özkan, Mertcan Gülben, Özdemir Şentürk, Emirhan Berberoğlu, Semra Türkan, Zeynep Öz, Ramazan Süleyman Göktürk, Hasan Akgül, Sinem Günaydın, Muhammet Murat Görgöz

**Affiliations:** ^1^ Faculty of Science, Department of Biology Akdeniz University Antalya Turkiye; ^2^ Faculty of Forestry, Department of Soil Science and Ecology Isparta University of Applied Sciences Isparta Turkiye; ^3^ Graduate School of Applied and Natural Sciences, Department of Biology Akdeniz University Antalya Turkiye; ^4^ Gölhisar Vocational School, Department of Forestry Burdur Mehmet Akif Ersoy University Burdur Turkiye; ^5^ Faculty of Literature, Department of Geography Akdeniz University Antalya Turkiye; ^6^ Faculty of Science, Department of Statistics Hacettepe University Ankara Turkiye

**Keywords:** GAM model, habitat filtering, mosaic habitats, Rao's quadratic entropy, topographic position index

## Abstract

This study investigates the variation in functional diversity (FD), species diversity (SD), and endemicity across all Mediterranean‐type vegetation belts (MVB) within a topographically complex mountainous refuge, focusing on their relationships with topographic and climatic factors. Microclimatic processes and mosaic habitats caused by topographic complexity increase the plant diversity of the area. This diversity is reflected in our study through the representation of different habitat types. The relationship of functional and species diversity with environmental parameters was also modeled and mapped within this study conducted with approximately 5550 records of 1017 plant taxa from 136 study plots. Functional diversity was measured using Rao's quadratic entropy, and alpha species diversity values were calculated using the Shannon‐Wiener index. Various regression models were trained and evaluated, and were assessed based on several statistical metrics. The final model selection, the Generalized Additive Model (GAM), was chosen based on its superior performance, ensuring the model not only fits the data well but also accurately predicts new data, thus optimizing both the validity and practical utility of the model. Our GAM results indicated that elevation is the most influential factor on diversity values, and that functional and species diversity curves show different trends with increasing elevation. Additionally, the topographic position index was identified as the most significant process affecting functional diversity in terms of “habitat filtering”. In this context, the variation in functional diversity, species diversity, and endemism in mosaic habitats creates a mosaic of diversity. The results emphasize the importance of topographic complexity in maintaining biodiversity and highlight the need for targeted conservation strategies that prioritize areas with high functional diversity, such as the Meso‐, Thermo‐, and Supra‐ Mediterranean vegetation belts, alongside habitats with high endemicity, particularly in the Oro‐ and Cryoro‐ vegetation belts.

## Graphical abstract

Within the scope of our study, we modeled and mapped the relationships between functional diversity (Rao's Q), alpha species diversity, and endemism with topographic and climatic factors in a mountainous refugial area encompassing all Mediterranean vegetation strata. To capture the diversity mosaic of these mosaic habitats, we classified our sample plots according to habitat types. While constructing data matrices, we evaluated the vegetative and regenerative traits of all seed plant species present in the study plots. Using a dataset comprising 5505 records of 1017 plant taxa from 136 sample plots, we completed the modeling and mapping processes.

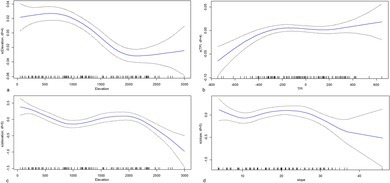

## 1 Introduction

The Mediterranean Basin ranks as the third most biodiverse region globally, following the Tropical Andes in South America and Sundaland in Southeast Asia (Myers et al. 2000), with nearly 22,500 plant species, most of which are endemic (Mittermeier et al. 2011). The presence of high mountains surrounding the Mediterranean, which host refuge areas with microclimates and a variety of different habitat types, contributes to the region's rich plant diversity and endemism (Väre et al. 2003; Ruiz‐Labourdette et al. 2011; Spehn et al. 2011). Mountain ecosystems in the Mediterranean region, both in Europe (Thuiller et al. 2005; Evangelista et al. 2016) and Anatolia (Médail and Quézel 1997; Parolly 2015), stand out as areas with high diversity. Nonetheless, these ecosystems are expected to experience significant changes due to climate change (Engler et al. 2011), posing threats to all Mediterranean mountain ecosystems (Pauli et al. 2012; Evangelista et al. 2016). Mountain ecosystems play a pivotal role in biodiversity conservation thanks to the interdependence of species, which ensures ecosystem stability through functional redundancy and enhances ecosystem functionality via the complementarity of traits and efficient resource utilization among taxa (Körner 2004, 2007). Within this context, mountain ecosystems serve as exceptional models for exploring how environmental factors influence the distribution and diversity of plant species or traits (Bruun et al. 2006; Gutiérrez‐Girón and Gavilán 2013; Chauvier et al. 2021; Vetaas 2021). In recent years, the number of studies conducted on functional diversity in mountainous areas of the Mediterranean Basin has increased in order to gain more detailed knowledge about ecosystem processes (e.g., Matteodo et al. 2016; Stanisci et al. 2020; Bricca et al. 2021).

In the most general terms, functional diversity is defined as the distribution of trait values in a community (Díaz and Cabido 2001; Tilman 2001), and provides principles and tools to establish connections between the characteristics of communities and ecosystem functions and services (Cornelissen et al. 2003; Díaz et al. 2007). The functional diversity approach holds significant potential to address questions such as the distribution of organisms along environmental gradients (Grime 1979; Westoby et al. 2002), the identification of rules governing community assembly (McGill et al. 2006; Shipley 2010; Suding et al. 2008), the translation of organismal functions into ecosystem‐level effects (Lavorel and Garnier 2002; Reich et al. 1992; Naeem and Wright 2003; Valencia et al. 2015; Peacador et al. 2015), and the understanding of how ecosystems regulate certain services beneficial to humanity (Díaz et al. 2007; Naeem et al. 2012). Environmental gradients, such as topographic factors—elevation, aspect, slope (Chapman and McEwan 2018), topographic position index (Bátori et al. 2023), topographic wetness index (Sørensen et al. 2005), etc., and climatic variables—annual mean temperature, mean diurnal range, isothermality, temperature seasonality, maximum temperature of the warmest month, etc. (Lee and Chun 2016; Naka et al. 2020) act as filters that can shape species assemblages by selecting specific functional traits and indices (Botta‐Dukát and Czúcz 2016). Therefore, topographic complexity creates an environmental mosaic, enabling species with diverse ecological needs to coexist within confined areas (Badgley et al. 2017). Understanding these gradients allows us to discern the mechanisms behind the observed patterns of functional diversity and helps predict how these communities may shift in response to environmental changes (de Bello et al. 2021; Garnier et al. 2016).

Habitat filtering, limiting similarity, niche specialization, and competition are fundamental processes that shape trait variation in plant communities (MacArthur and Levins 1967; Zobel 1997; Mason et al. 2005; Violle et al. 2012). It is widely assumed that community assembly operates under the combined influence of these four mechanisms (de Bello et al. 2021; Götzenberger et al. 2012). Habitat filtering selectively favors species with traits better adapted to local environmental conditions, resulting in trait convergence as it reduces trait variation by excluding unsuitable trait values (Weiher et al. 1998). In contrast, limiting similarity prevents the coexistence of species with highly similar traits through interspecific competition, leading to trait divergence as it increases trait variation by forcing coexisting species to occupy distinct niches and to be spaced out in trait space (Götzenberger et al. 2012). Niche specialization acts as a complementary force, further driving trait divergence by enabling species to exploit distinct ecological niches and reducing interspecific competition (D'Andrea and Ostling 2016). This mechanism allows species to persist in shared environments by specializing in particular resources or conditions, promoting functional diversity. In parallel, competition influences trait variation by limiting the similarity of coexisting species through selective pressures on overlapping niches, as described in classical niche theory (MacArthur and Levins 1967; Chesson 2000). These interactions highlight the multidimensional nature of niche axes and their critical role in structuring trait variation within plant communities (D'Andrea and Ostling 2016). The interplay of these processes can sometimes balance each other, resulting in no significant departure from randomness in trait distributions (Botta‐Dukát and Czúcz 2016), and is strongly dependent on the environmental heterogeneity of the datasets studied (de Bello 2012).

As is well known, there are many different approaches to calculating functional diversity: dissimilarity matrices, multidimensional spaces, functional dispersion, etc. (Schleuter et al. 2010; Mammola et al. 2021). Rao's quadratic entropy (Rao's Q), one of the dissimilarity matrix approaches, is highlighted as an effective measure because it combines species abundances and trait differences, providing a robust framework for understanding functional diversity (Botta‐Dukát 2005). It has been used in various ecological studies to model community‐environment relationships and assess the impact of biodiversity on ecosystem functioning. Additionally, Rao's quadratic entropy appears to be the most suitable metric for detecting both trait convergence, driven by “habitat filtering”, and trait divergence, resulting from “limiting similarity” (de Bello 2012; Botta‐Dukát and Czúcz 2016; de Bello et al. 2021). Botta‐Dukát (2005) stated that FD Rao's Q is influenced by both species abundance‐based diversity and differences among species, meaning that it can decrease even when species richness increases. The reason for this is that functional diversity is not solely determined by species richness, but also by the distribution of functional traits within the community. The introduction of a new species into a community increases species richness but may reduce functional diversity if the newly introduced species is functionally redundant with existing ones (Botta‐Dukát 2005). In recent years there has been an increase in studies combining functional diversity with complex null models (e.g., Moradi and Oldeland 2019; Bricca et al. 2019). These models compare observed values with randomly generated expected community values. There is a consensus on using the standardized effect size (SES) of functional diversity to measure the magnitude of assembly processes (de Bello 2012; Botta‐Dukát 2018). However, functional diversity indices that are not strongly related to species richness can provide results comparable to those obtained through null models, particularly when analyzed along environmental gradients such as altitude (de Bello 2012; de Bello et al. 2021). In this study, we aimed to determine the most realistic plant diversity within the sample plots, ensuring that our dataset accurately reflects the floristic composition of the study area. Since our dataset is based on a comprehensive inventory of seed plant diversity rather than incomplete sampling, we used FD Rao's Q, which directly quantifies functional diversity without relying on null model assumptions. Furthermore, we found no significant correlation between species richness and FD Rao's Q, indicating that functional diversity in our dataset was primarily shaped by the distribution of functional traits within communities rather than by species richness alone. We evaluated the relationship between functional diversity and climatic as well as topographic features across the montane Mediterranean vegetation belts (MVB). Our study area, encompassing a range from near sea level to mountain peaks and characterized by its complex karstic topography that supports diverse habitat types, provided an opportunity to analyze the relationships among the mosaic habitats shaped by the Mediterranean climate. Additionally, we calculated species diversity to better evaluate trait convergence and divergence, and endemicity to identify more sensitive areas; both metrics were subsequently used to compare with functional diversity. We determined functional diversity using Rao's Q, which is unaffected by species diversity, and alpha species diversity using the Shannon‐Wiener index, which incorporates abundance values. We modeled functional and species diversity and made efforts to determine the best model representing the relationship between them and environmental factors. Using the best‐performing model, we created maps for both diversity indices to visualize variations in diversity across the mountainous ecosystem. Our study primarily aimed to answer the following questions:

Q1: Rao's Q, alpha species diversity and endemicity exhibit distinct patterns of variation with increasing elevation. What is the relationship between these metrics in Mediterranean vegetation belts? Q2: How do Rao's Q and alpha species diversity values in complex Mediterranean ecosystems change with increasing elevation? Q3: In mountainous Mediterranean ecosystems, where microclimate strongly influences diversity, topographic factors are among the most important drivers of biodiversity. In these ecosystems, which topographic factor(s) stand out in terms of “habitat filtering”? Q4: The variation in diversity metrics in mosaic habitats creates a mosaic of diversity. How do Rao's Q, alpha species diversity, and endemicity values change in mosaic habitats?

## 2 Material and Methods

### 2.1 Study Area and Data Collection

**FIGURE 1:**
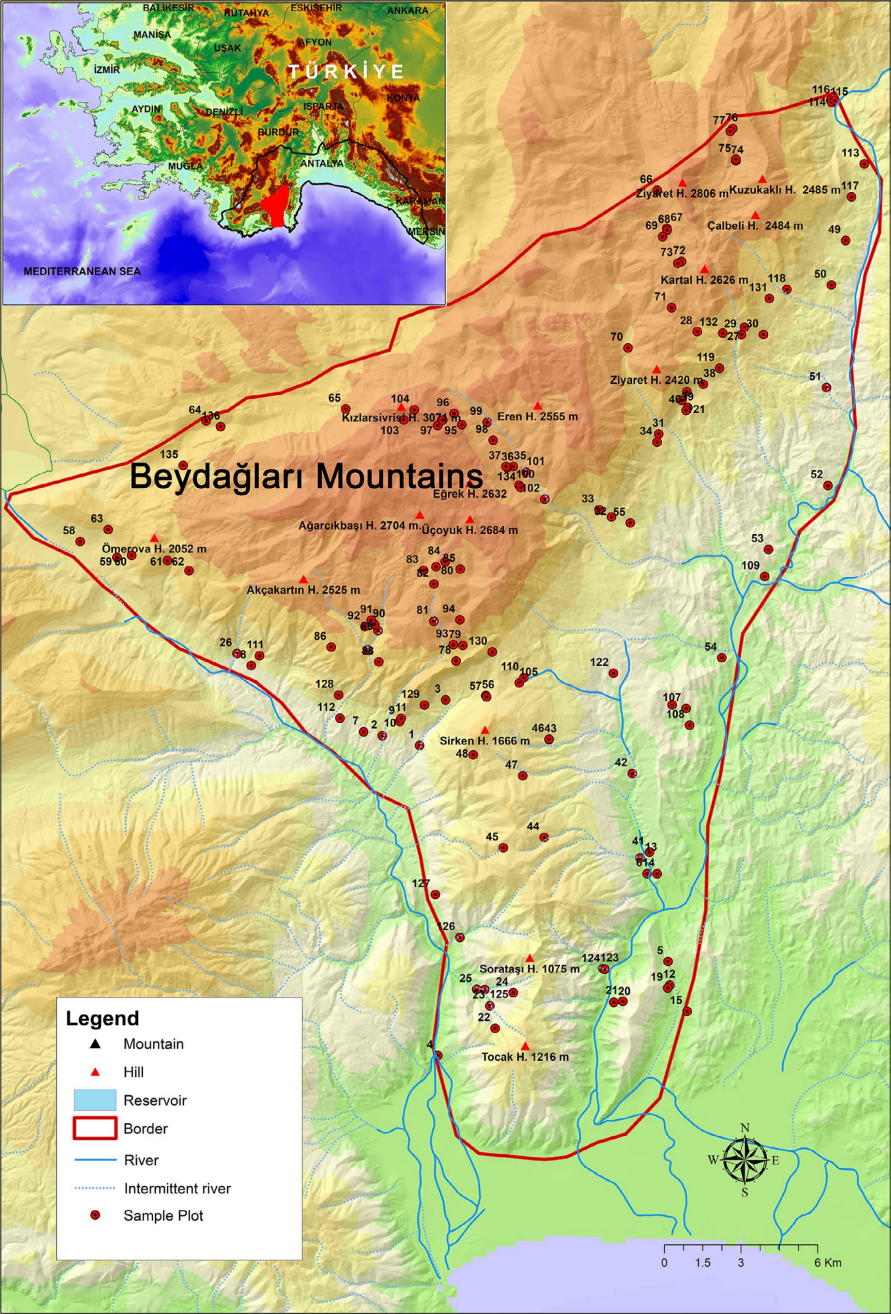
Topographic map of the study area with all sample plots.

Our study area is located in the Western Taurus Mountains, which are among the richest areas in terms of endemism in Turkey (Parolly 2015; Noroozi et al. 2019) and are considered a putative refuge area according to Médail and Diadema (2009). The region, isolated by valleys formed by two rivers flowing into the Mediterranean—the Akçay to the west and the Alakır to the east—rises from the coastal regions and extends northward. About 34% of the region lies within the high mountain belt, where the Bey Mountains, reaching a peak elevation of 3087 m, are aligned from west to east in the northern section. Additionally, it encompasses the Sarıkaya Wildlife Development Area and also includes the Dibek Nature Conservation Area, which is home to the world's oldest cedar tree. The substantial coverage of protected areas within the study area is crucial for the observation of natural systems. The topographic map showing the location and boundaries of the study area with all sample plots is presented in Figure 1.

The geomorphological units within the study area exhibit variation influenced by elevation, geology, and climate. At higher elevations, features shaped by glacial activity, such as cirques, dominate, alongside karstic dissolution forms like uvalas and dolines. As the elevation decreases, the landscape transitions to topographies sculpted by fluvial activity. Overall, the study area is characterized by geomorphological units including glacial forms, karst features, steep slopes, slope debris, narrow and deeply incised valleys, and riverbeds. The floristic composition of the area has been sampled to represent a variety of habitat types along different vegetation belts in the study, and in such complex ecosystems, the sample size and number are significant, and their adequacy should be tested. Therefore, preliminary field studies were conducted to determine the minimum sample area size that best represents the entire area, and the sample area adequacy was determined to be 30 × 30 m (for further information, see Özkan et al. 2023). Additionally, to determine whether the number of sample areas is sufficient, the jackknife procedure (Heltshe and Forrester 1983) was employed, and the calculations were made according to the Formula (1) provided below.

(1) $$S_{j}^{*}=S_{\mathrm{obs}}+Q_{1}\left(\frac{Q-1}{Q}\right)$$

In the equation, $S_{j}^{*}$ represents the estimated species richness according to the jackknife index, $S_{\mathrm{obs}}$ represents the number of species determined by the inventory, Q represents the total number of sample areas, and $Q_{1}$ represents the number of species in a given sample plot. Guided by the criterion established by Munguía‐Rosas and Montiel (2014), which states that achieving 80% of estimated species richness is sufficient, we continued our sampling until this percentage was attained. The 80% threshold was reached when we had collected data from 136 sample plots.

### 2.2 Sampling of Vegetation Belts With Their Variety of Habitats

**FIGURE 2:**
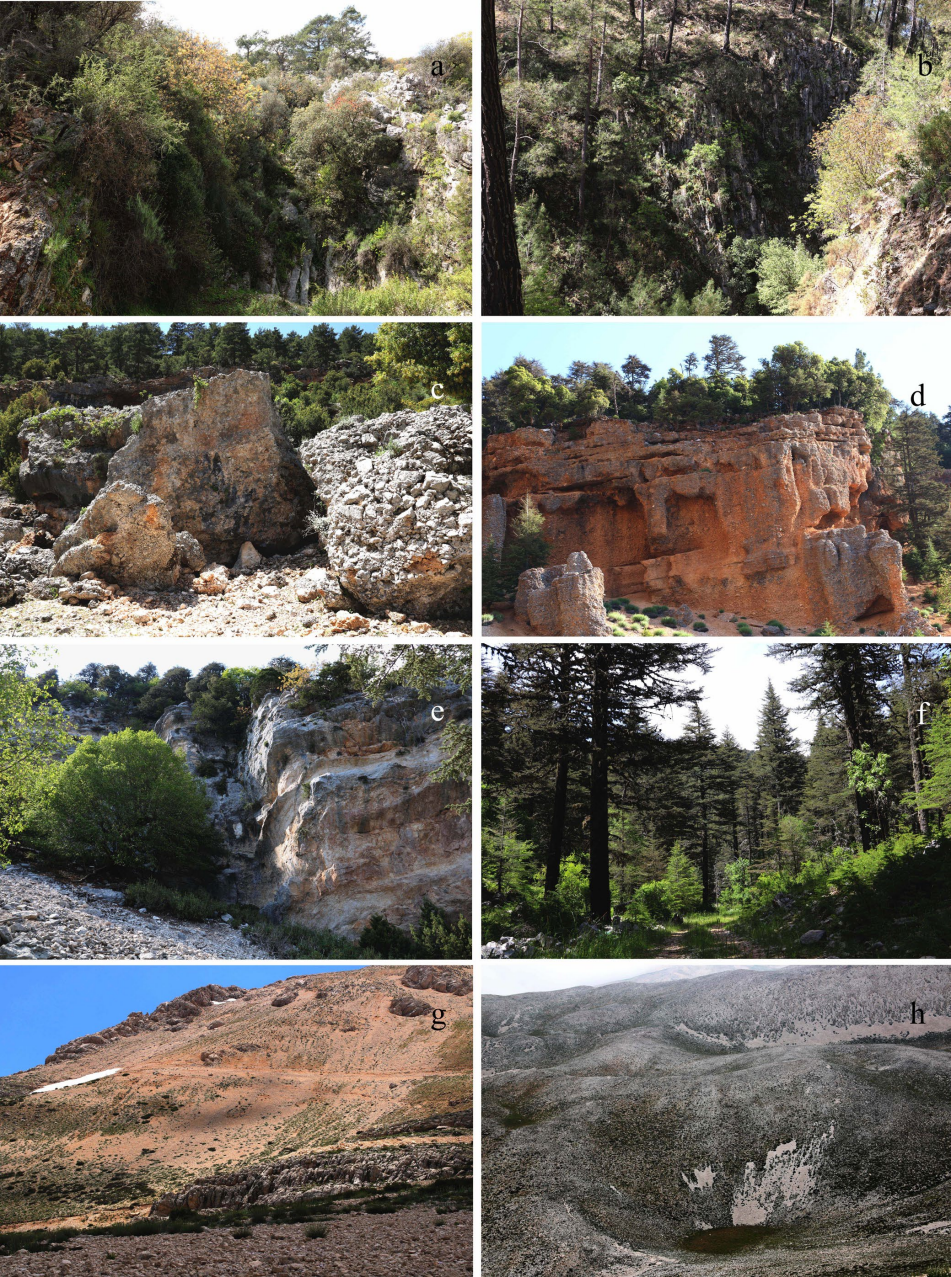
Photographs of habitat types taken from different vegetation belts in the study area. (a) Rocky areas dominated by shrub vegetation and (b) valleys within *Pinus brutia* forest in Thermo‐MVB; (c, d) a mosaic of scree, rocks, shrubs, and forests in Meso‐ and Supra‐MVB, respectively; (e) steep cliffs characterized by talus deposits and (f) *Cedrus libani* forests in Oro‐MVB; (g) a mosaic of scree, rocks, and stony steppes (g) and (h) dolines in Cryoro‐MVB.

Our study area represents all Mediterranean vegetation belts (MVB) with an elevation range from 54 to 3070 m, featuring mosaic habitats, where topographic variability creates diverse habitat conditions that support biodiversity. In the Illustrated Flora of Turkey, Ketenoğlu et al. (2014) classified the vegetation belts of the mountainous areas in Turkey's Mediterranean region into 500‐m intervals. This classification has also been adopted in our study: the 0–500 m range is designated as the Thermo‐Mediterranean Vegetation Belt (MVB), 500–1000 m as the Meso‐MVB, 1000–1500 m as the Supra‐MVB, 1500–2000 m as the Oro‐MVB, and elevations above 2000 m as the Cryoro‐MVB. In the study, out of the 136 designated study plots, 27 are in the Thermo‐MVB, 26 are in the Meso‐MVB, 26 are in the Supra‐MVB, 21 are in the Oro‐MVB, and 36 are in the Cryoro‐MVB. Within the scope of the study, study plots were grouped based on habitat types, which were classified according to vegetation cover and landform characteristics based on expert observations. This classification identified a total of eight distinct habitat types, including forests (closed and semiclosed forests), shrublands, degraded areas, rocks, screes, steppes, and dolines. As is generally accepted, forests with canopy coverage between 71% and 100% are classified as closed forests, while those with canopy coverage between 41% and 70% are classified as semiclosed forests (Kaptan 2021). The distribution of sample plot numbers across vegetation zones and habitat types is provided in Table 1. The photographs of habitat types taken from different vegetation belts in the study area are presented in Figure 2.

**TABLE 1:** Number of sample plots according to vegetation belts and habitat types.

Vegetation belts	Closed forests (1)	Semiclosed forests (2)	Shrublands (3)	Degraded areas (4)	Rocky vegetation (5)	Screes (6)	Steppes (7)	Dolines (8)	Total
Thermo‐MVB	5	4	10	1	6	1	—	—	27
Meso‐MVB	3	6	4	1	9	3	—	—	26
Supra‐MVB	6	9	2	—	4	5	—	—	26
Oro‐MVB	6	4	—	1	1	2	7	—	21
Cryoro‐MVB	—	—	—	—	1	4	26	5	36
Total	20	23	16	3	21	15	33	5	136

During field studies conducted ad hoc for this study between March 2021 and September 2023, totally 1017 different plant taxa were identified from all study plots, 174 of which are endemics, and 5505 records were obtained for these taxa. Within the scope of the study, all members of the Spermatophyta in the sample plots were recorded and each sample plot was visited in different flowering periods. Coverage of the species within each plot was arranged to correspond to a value between 1 and 9 according to Westhoff and Van Der Maarel (1978) (Appendix S1). The plant specimens were primarily identified using the “Flora of Turkey and the East Aegean Islands” (Davis 1965–1985; Davis et al. 1988; Güner et al. 2000). Additionally, some specimens were identified using reference works on floras from neighboring countries such as the “Flora Europaea” (Tutin et al. 1964–1980) and “Flora Iranica” (Rechinger 1965–1977), as well as flora and vegetation studies conducted in areas close to the study area, and various revision studies completed after the writing of the Flora of Turkey. The scientific names and endemism information of taxa were organized using the “World Flora Online” (WFO 2025), the “World Checklist of Vascular Plants” (WCVP 2025), and the “List of Turkish Plants” (Güner et al. 2012).

### 2.3 Functional Traits

The process of selecting appropriate plant functional traits is crucial for accurately measuring functional diversity within ecosystems. Despite this importance, there is no consensus among researchers regarding which traits should be chosen and the number of traits to be used (Cornelissen et al. 2003; Pla et al. 2012; Pérez‐Harguindeguy et al. 2013; Garnier et al. 2016; de Bello et al. 2021). This selection can vary depending on the purpose of the study, the number of species included in the study, the size of the study area, and the specific goals aimed to be achieved (Garnier et al. 2016). We evaluated a total of 8 functional traits which are related to vegetative and regenerative characteristics, namely “life form”, “clonality”, “nutrient uptake strategy”, “spinescence”, “leaf phenology”, “flowering period”, “floral longevity” and “dispersal strategy”. These traits do not require hard laborious measurement processes but can provide highly informative insights into ecosystem processes (see Cornelissen et al. 2003). The subcategories of the traits were determined according to Cornelissen et al. (2003) and Pérez‐Harguindeguy et al. (2013), and are presented in Table 2. Some taxa may fit into more than one subcategory of a particular trait. The traits of taxa were determined.

**TABLE 2:** List of the plant traits and their categories utilized in the species by traits matrix. $\left(-\right)$ means excluded.

Trait	Trait categories
Life form	Phanerophytes (1), chamaephytes (2), hemicryptophytes (3), cryptophytes (4), hydrophyte (5), therophyte (6), vascular parasite (7)
Clonality	Nonclonal (1), rhizomes (2), roots (3), indistinguishable rhizome or root (4), bulb, corm or tuber (5), stolon (6), turion (7)
Nutrient uptake	Nitrogen fixers (1), ectomycorrhizae (2), arbuscular mycorrhizae (3), ericoid mycorrhizae (4), orchid mycorrhizae (5), hemiparasites (6), mycoheterotrophs (7), holoparasites (8), nonmycorrhizal plants (9)
Spinescence	No spines (0), low density of soft spines < 5 mm (1), high density of soft spines or intermediate hardness spines (2), high density of hard spines > 5 mm (3), high density of hard spines > 20 mm (4), high density of hard spines > 100 mm (5), leaf tips ending in spines (6)
Leaf phenology	Evergreen (1), partially deciduous (2), completely deciduous (3) − herbaceous
Floral longevity	Each taxon was quantified by recording the number of months the plant remained in flower − gymnosperm
Flowering duration	1, 2, 3, …, 10, 11, 12 − gymnosperm
Dispersal strategy	Autochory (1), anemochory (1), endozoochory (2), ectozoochory (3), dispersal by hoarding (4), myrmecochory (4), hydrochory (5), ballistochory (6), no dispersal (7)

Based on descriptions in the “Flora of Turkey and the East Aegean Islands” (Davis 1965–1985; Davis et al. 1988; Güner et al. 2000), the Pladias (Chytrý et al. 2021) and BROT 2.0 (Tavşanoğlu and Pausas 2018) databases when descriptions were insufficient, as well as various revision and monograph studies, field observations, and expert opinions. The data matrix containing the traits of taxa is presented in Appendix S1.

### 2.4 Quantitative Diversity Analysis Methods

Within the scope of this study, functional diversity (FD) was calculated using Rao's Q index Formula(2), which is a generalized form of the Simpson diversity index (Botta‐Dukát 2005; Ricotta 2005; de Bello et al. 2009; Özkan 2023).

(2) $${\mathrm{FD}}_{\mathrm{Rao}}=\sum_{i=1}^{S}\sum_{j=1}^{S}p_{i}p_{j}d_{\mathrm{ij}}$$

In the formula, S represents the total number of species i and j, while *p*_*i*_ and *p*_*j*_ are the relative abundances of species i and j within the community, respectively. The dissimilarity between species i and j is denoted as *d*_*ij*_.

The analysis includes all calculated values from the dissimilarity matrix for each pair of species, ensuring that the dissimilarities for both S_1_ −S_2_ and S_2_ −S_1_ pairs are considered in the computations.

Gower's index, accommodating various traits even with missing data, can be extended to more variable types to calculate distances among species at different functional scales (Pavoine et al. 2009). The Gower distance Formula (3) (Gower 1971) was employed for the calculation of FD, as referenced in Podani (1999) and Podani and Schmera (2006).

(3) $${\mathrm{d}}_{k}=\frac{\sum_{i=i}^{n}w_{\mathrm{ijk}}s_{\mathrm{ijk}}}{\sum_{i=i}^{n}w_{\mathrm{ijk}}}$$

In the formula, *n* represents the number of functional traits, d_jk_ denotes the Gower dissimilarity value between species j and k, and $s_{\mathrm{ijk}}$, is the measure of discordance for functional trait i between species j and k.

The weight $w_{\mathrm{ijk}}$ is Boolean and is set to 0 if functional trait i is absent in both paired species (species j and k); it is set to 1 if functional trait i is present in either or both of the paired species. Additionally, $s_{\mathrm{ijk}}$ provides a measure of discordance for the variable i between species j and k.

Functional traits encompass various data types. Depending on the data type contained within these traits, the $s_{\mathrm{ijk}}$ values were calculated using different Formulas (4), (5), (6), (7), and (8).

The traits *(1) Life Form, (2) Clonality, (4) Leaf Phenology, (5) Nutrient Uptake, and (6) Dispersal Strategy* contain nominal data. For these traits, Formulas (4) and (5) were employed.

(4) $$\text{If}\;X_{\mathrm{ij}}\;\text{does not equal}\;X_{\mathrm{jk}},\text{then}\;s_{\mathrm{ijk}}=1,\text{indicating complete dissimilarity}$$

(5) $$\text{Conversely},\text{if}\;X_{\mathrm{ij}}\;\text{equals}\;X_{\mathrm{jk}},\text{then}\;s_{\mathrm{ijk}}=0,\text{indicating}\;\mathrm{no}\;\text{dissimilarity}$$

Among the functional traits, *(3) Flowering Duration* contains interval data. For this trait, Formula (6) was utilized.

(6) $$S_{\mathrm{iJk}}=x=\frac{|\mathrm{Xij}–\mathrm{Xik}|\;}{\text{Maximum}\;\left(\mathrm{Xi}\right)–\text{Minimum}\;\left(\mathrm{Xi}\right)}$$

Trait *8. Floral Longevity* contains ordinal data. For this trait, Formula (7) was employed.

(7) $$S_{\mathrm{iJk}}=x=\frac{|\mathrm{Xij}–\mathrm{Xik}|\;}{\text{Maximum}\;\left(\mathrm{ri}\right)–\text{Minimum}\;\left(\mathrm{ri}\right)}$$

While calculating dissimilarity using the Gower distance, all functional traits were equally weighted, and the distances between values were standardized to fall within a range of 0 to 1. Formula (8) was employed for this standardization.

(8) $$z=\frac{x-\text{minimum}\left(\;,x\right)}{\text{maximum}\left(\;,x\right)-\text{minimum}\left(\;,x\right)}$$

In Formula (8), x represents the actual value of the relevant species, $\min\left(x\right)$ denotes the value of the species with the smallest value in the sample area, and $\max\left(x\right)$ represents the value of the species with the highest value. The variable z in the formula provides the standardized value of the species. To facilitate these calculations, a custom macro was developed in Microsoft Excel. This macro automates the input, processing, and computation of FD indices using the formulas described above. The macro ensures consistency and accuracy in the analysis by standardizing the procedures and reducing the potential for manual errors.

Alpha species diversity (SD) values were calculated using the Shannon–Wiener index (9; Shannon 1948). The calculations were performed using the Biological Diversity Components (BİÇEB) software (Özkan et al. 2020).

(9) $$H^{\prime }=-\sum_{i=1}^{S}P_{i}\cdot \log\left(p_{i}\right)$$

Where $H^{\prime }$ is the Shannon–Wiener index, S is the total number of species, and $p_{i}$ is the relative abundance value of species i.

To examine the relationship between diversity and endemicity values across different vegetation belts and habitat types, one‐way ANOVA and post hoc tests were performed by using IBM SPSS Statistics (Version 27). Moreover, to ensure comparability across different metrics (Species Diversity, Functional Diversity, and Endemicity), we standardized the data using Min‐Max scaling for box plots. The Min‐Max scaling was performed using the *sklearn.preprocessing* module in Python (Pedregosa et al. 2011). These plots were created using the *matplotlib* (Hunter and Dale 2007) and *seaborn* (Waskom 2021) libraries in Python, which offer robust and flexible plotting capabilities.

### 2.5 Climatic and Topographic Variables

Initially, a Digital Elevation Model (DEM) was obtained based on the study area boundaries from the USGS (2025). Elevation variables were derived from the DEM at a grid size of 100 × 100 m through *ArcGIS 10.8* software. Subsequently, from the elevation variable, the aspect and slope variables were created. The “*Topographic Tools*” plugin was employed to develop the Topographic Position Index (TPI), the Topographic Wetness Index (TWI) for identifying moist or dry areas, and the Solar Illumination Index (sollidx) to indicate sunlight exposure at various times of the day, all based on the elevation variable. The Radiation Index (radidx) variable was produced using the “*Geomorphometry and Gradient Metrics Toolbox*” plugin, based on the aspect variable. This radiation index varies between 0 and 1, with transformation values of “0” in the north‐northeast direction, and “1” on the warmer and drier south‐southwest slopes (Roberts and Cooper 1989). The geological map of the study area was obtained from the General Directorate of Mineral Research and Exploration of Türkiye (MTA). After utilizing the geometric data, each bedrock type within the study area boundaries was digitized into polygons, and an attribute table was subsequently created. Based on the rock types listed in the attribute table, a layer was created in vector format, exported at a grid size of 100 × 100 m into raster format, and variables for rock types (Limestone: Lime, Sandstone: Sands, Pebble: Pbst, Chert: Chrt, Alluvium: Alluv, Volcanite: Volc, Ophiolite: Ophi, Basalt: Baslt, Claystone: Clayst) were generated. For land classification, the *Corine Land Cover* map, which includes 15 different categories and was created under the Corine project conducted by the European Environment Agency (EEA), was utilized (Bossard et al. 2000). For further details, see Appendix S3.

In the second phase, the production of climate variables was initiated. In this context, climate variables were freely downloaded from the *CHELSA* climate database available at https://chelsa‐climate.org (Karger et al. 2017). Nineteen bioclimatic variables (Bio 1–19) with a cell size of 30 arc‐seconds (~1 km) available in the *CHELSA* database were used. The downloaded 19 bioclimatic variables were resampled in *ArcGIS 10.8* software to match the same dimensions (100 × 100 m) as the environmental variables (Appendix S2).

### 2.6 Statistical Modeling

**FIGURE 3:**
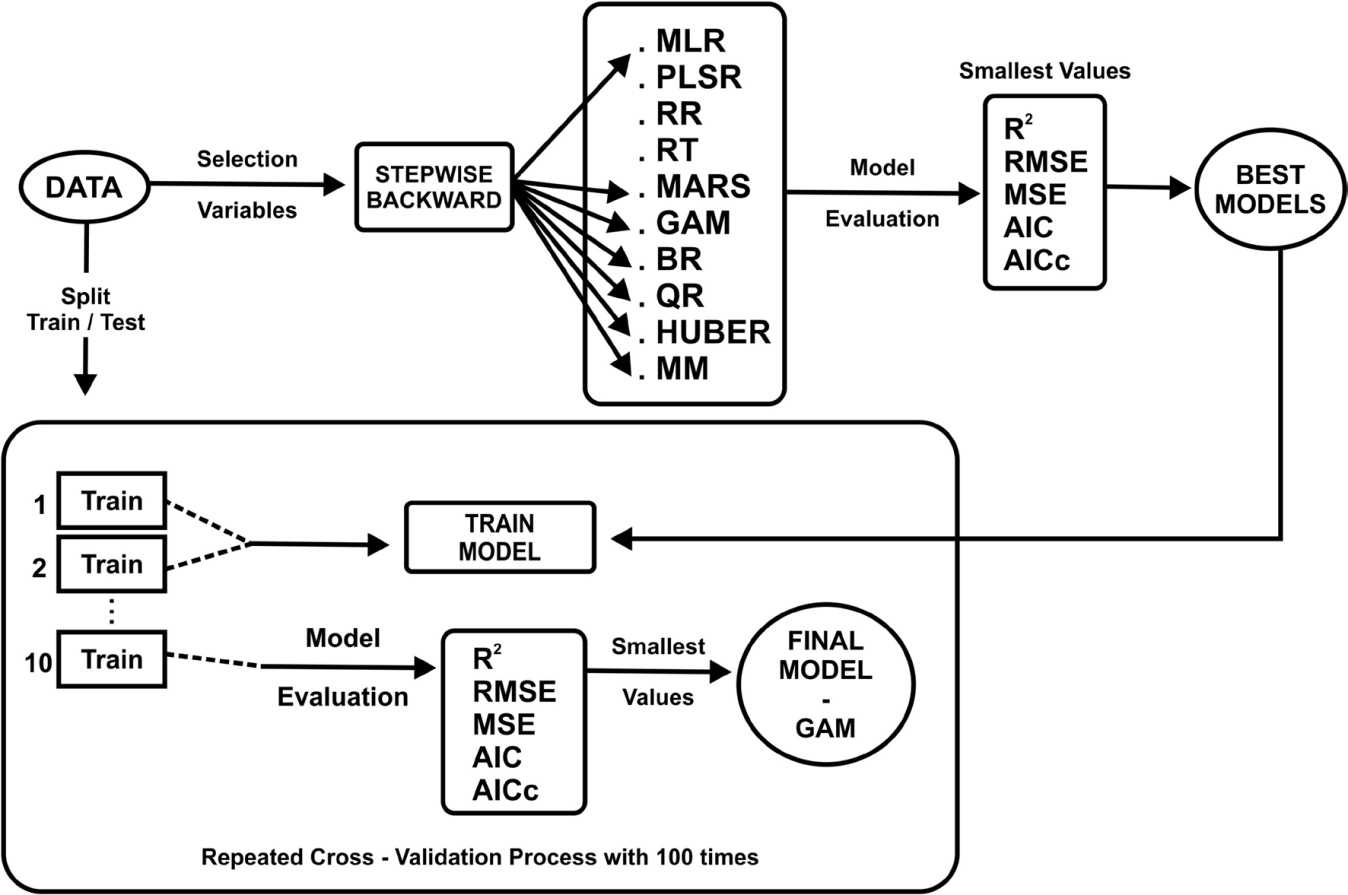
Workflow diagram of diversity modeling processes. AIC, Akaike Information Criterion, AICc, Corrected Akaike Information Criterion; BR, beta regression; GAM, generalized additive model; HUBER, huber regression; MARS, multivariate adaptive regression splines; MLR, multiple linear regression; MM, MM‐estimators; MSE, mean squared error; PLSR, partial least squares regression; QR, quantile regression; *R* ^2^, *R*‐squared or the coefficient of determination; RMSE, root mean squared error; RR, ridge regression; RT, regression tree.

In this study, the relationship between FD values and environmental factors was modeled using various statistical methods. These methods included Multiple Linear Regression (MLR), Partial Least Squares Regression (PLSR), Ridge Regression (RR), Regression Tree (RT), Multivariate Adaptive Regression Splines (MARS), Generalized Additive Model (GAM), Beta Regression (BR), Quantile Regression (QR), Huber Regression, and MM‐estimators. The modeling steps are summarized in Figure 3. As shown in the workflow diagram (Figure 3), the first step was variable selection, which was done using “stepwise” or “backward” methods. R packages such as “MuMIn,” “pls,” “lmridge,” “rpart,” “earth,” “gam,” “betareg,” “quantreg,” and “MASS” were used for this purpose. Variables were selected based on the Akaike Information Criteria (AIC). The models were then evaluated using 10‐fold cross‐validation to prevent overfitting, especially for complex models like GAM and MARS. Model performance was tested using metrics such as *R* ^2^, MAE, RMSE, MSE, AIC, and AICc, and each model was repeated 100 times during the cross‐validation process.

According to the results summarized in Table 3, the GAM model had the lowest error metrics (MAE, RMSE, MSE), the smallest AIC and AICc values, and the highest *R* ^2^ values on the test dataset. Therefore, GAM was chosen as the best‐performing model. GAM was also used to model the relationships between alpha species diversity values and environmental variables to enable comparisons with FD.

**TABLE 3:** Results of training and test datasets regarding the models used in modeling FD diversity components. Bold values indicate the best‐performing model (GAM), which achieved the highest *R* ^2^ and the lowest MAE, RMSE, and most negative AICc values, indicating high prediction accuracy and model stability.

	*R* ^2^		MAE		MSE		RMSE		AIC		AICc	
	Training	Test	Training	Test	Training	Test	Training	Test	Training	Test	Training	Test
MLR	0.306	0.267	0.024	0.024	0.001	0.001	0.030	0.031	−949.329	−388.412	−949.239	−388.317
PLSR	0.282	0.260	0.024	0.025	0.001	0.001	0.031	0.031	−946.736	−389.983	−946.706	−389.952
RR	0.295	0.238	0.025	0.026	0.001	0.001	0.031	0.032	−941.826	−386.180	−941.736	−386.086
RT	0.456	0.276	0.020	0.024	0.001	0.001	0.028	0.031	−978.497	−388.125	−978.191	−388.031
MARS	0.515	0.188	0.019	0.025	0.001	0.001	0.025	0.036	−982.055	−364.486	−980.295	−363.803
GAM	**0.446**	**0.344**	0.021	**0.023**	0.001	0.001	0.027	**0.029**	−979.598	**−394.601**	−979.508	−**394.506**
BR	0.283	0.260	0.024	0.025	0.001	0.001	0.031	0.031	−946.752	−389.983	−946.722	−389.952
QR50	0.282	0.261	0.024	0.025	0.001	0.001	0.031	0.031	−946.035	−389.194	−946.005	−389.163
QR75	0.282	0.263	0.031	0.031	0.002	0.002	0.039	0.040	−877.031	−362.847	−877.001	−362.815
HUBER	0.282	0.262	0.024	0.025	0.001	0.001	0.031	0.031	−946.325	−389.893	−946.295	−389.862
MM	0.282	0.263	0.024	0.025	0.001	0.001	0.031	0.031	−946.144	−389.853	−946.114	−389.822

Bold values indicate the best‐performing model (GAM), which achieved the highest R² and the lowest MAE, RMSE, and most negative AICc values, indicating high prediction accuracy and model stability.

Subsequently, mapping was performed for FD and SD based on the variables structuring the models, using a distribution matrix with a resolution of 100 × 100 m cells. The base maps were created to represent the topography of the study area. For an exhaustive account of the modeling processes and detailed results, refer to Appendix S3.

## 3 Results

From all sample plots, 1017 plant taxa (974 species) belonging to 92 different families and 409 genera were identified, with approximately 17% being endemic. Floristic composition and habitat types were assessed by representing them with sample plots. In the Thermo‐MVB, a total of 440 taxa were recorded, 31 of which are endemic; in the Meso‐MVB, there were 388 taxa, including 47 endemics; in the Supra‐MVB, there were 446 taxa, including 66 endemics; in the Oro‐MVB, there were 393 taxa, including 89 endemics; and in the Cryoro‐MVB, there were 313 taxa, including 88 endemics.

### 3.1 Relationship of Dependent Variables With Vegetation Belts and Habitat Types

**FIGURE 4:**
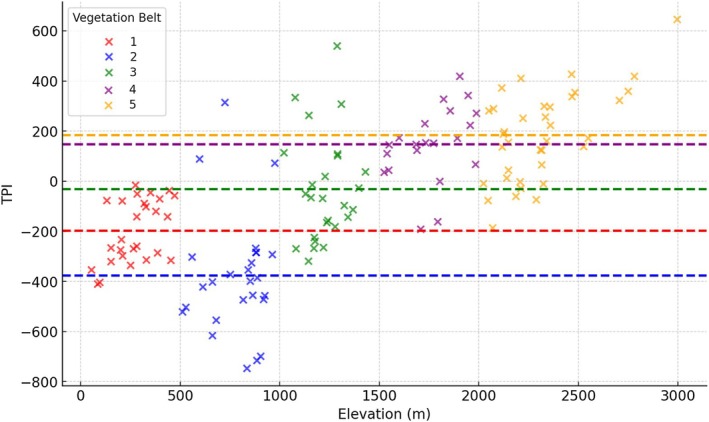
Scatter plot showing the distribution of TPI values of the study plots across elevation. Each point represents a study site, color‐coded by vegetation belt. The dashed lines indicate the mean of TPI values for each vegetation belt.

The explanatory variables (response data) in this study are FD, SD, and endemicity. A Pearson correlation analysis was conducted to evaluate the statistical relationships between these variables. According to the obtained results, a significant negative correlation is observed between endemicity and FD (*r* = −0.46, *p* < 0.01), while a moderate negative correlation is found between endemicity and SD (*r* = −0.29, *p* < 0.05), and a nonsignificant relationship is detected between FD and SD (*r* = 0.12, *p* > 0.05). Sample plot number (n) for all correlations is 136. Significance levels are indicated by *p*‐values, with a threshold of 0.05 for statistical significance. Our ANOVA analysis results show statistically significant differences among vegetation belts for FD, SD, and endemicity. The post hoc test demonstrates that Thermo‐, Supra‐, and Meso‐MVBs differ significantly from the Oro‐ and Cryoro‐MVBs in terms of FD. The highest mean FD values are observed in the Thermo‐, Supra‐, and Meso‐MVBs, while significantly lower values are detected in the Oro‐ and Cryoro‐MVBs. Species richness exhibits a different distribution among vegetation belts compared to FD. The post hoc test results show that the Thermo‐MVB, with the highest mean SD, is significantly different from all other belts except the Oro‐MVB. Interestingly, the Oro‐MVB, which ranks second in SD after the Thermo‐MVB, has the lowest mean FD among the belts. On the other hand, endemicity increases from lower to higher elevations. The post hoc test reveals that the Thermo‐MVB has a statistically significantly lower endemicity compared to other vegetation belts. The endemicity of the Meso‐ and Supra‐MVBs is found to be significantly higher than that of the Thermo‐MVB but significantly lower than that of the Oro‐ and Cryoro‐MVBs. Conversely, the endemicity of the Oro‐ and Cryoro‐MVBs is statistically significantly higher than that of the other three belts. The post hoc test results for pairwise comparisons of MVBs (for detailed results see Appendix S3), along with the elevation and mean TPI values of the study sites within each vegetation belt, are presented in Table 4. Additionally, the variation of TPI values with elevation and their distribution across vegetation belts are illustrated as a scatter plot in Figure 4. TPI exhibits a positive correlation with elevation; however, each vegetation belt has distinct values influenced by its unique topography.

**TABLE 4:** Pairwise comparisons of MVBs based on post hoc test results, showing significant differences in FD, SD, and endemicity. Mean elevation and TPI values for sampled plots within each MVB are presented in parentheses.

MVB Alt (mean); TPI (mean)	1	2	3	4	5
Thermo‐MVB (1) Alt (275 m) TPI (−198)		SD ↑ E ↓	SD ↑ E ↓	FD ↑ E ↓	FD ↑ SD ↑ E ↓
Meso‐MVB (2) Alt (786 m) TPI (−377)	SD ↓ E ↑		—	FD ↑ E ↓	E ↓
Supra‐MVB (3) Alt (1226 m) TPI (−32)	SD ↓ E ↑	—		FD ↑ E ↓	FD ↑ E ↓
Oro‐MVB (4) Alt (1762 m) TPI (+147)	FD ↓ E ↑	FD ↓ E ↑	FD ↓ E ↑		—
Cryoro‐MVB (5) Alt (2310 m) TPI (−185; +184; +645)	FD ↓ SD ↓ E ↑	FD ↓ E ↑	FD ↓ E ↑	—	

**FIGURE 5:**
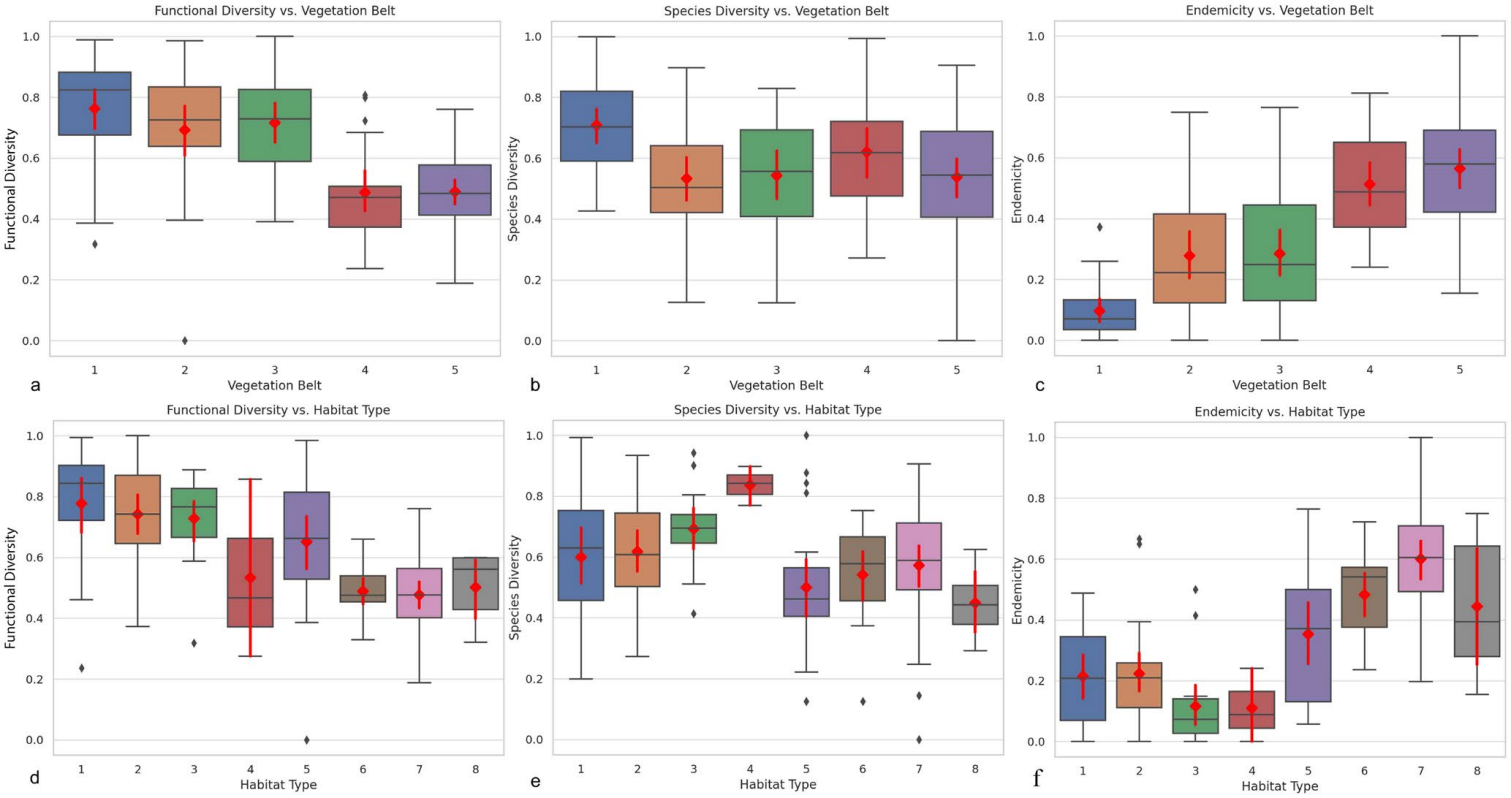
Box plots of diversity metrics by vegetation belts and habitat types. (a) Functional diversity across vegetation belts, (b) Species diversity across vegetation belts, (c) Endemicity across vegetation belts, (d) Functional diversity across habitat types. (e) Species diversity across habitat types, (f) Endemicity across habitat types. Each box plot includes the median (red line) and the interquartile range, with red diamonds representing the mean values.

Our ANOVA results and Figure 5 indicate that forests sampled from all other vegetation belts excluding the high mountain belt are the habitat types with the highest average FD. Closed forests have higher average FD and lower average SD compared to semiclosed forests. Among all habitat types, degraded areas, shrublands, and forests have the lowest endemism rates, respectively. In terms of average FD values, habitat types other than forests are ranked from highest to lowest as follows: shrublands, rocky vegetation, degraded areas, dolines, screes, and steppes. Within the scope of the study, degraded areas were sampled with one plot each from Thermo‐, Meso‐, and Oro‐MVBs, where forest degradation is most common. Although these areas have the highest average SD, they have the lowest of both FD average and endemicity within their vegetation belts according to our results. Rocky vegetation has low SD values but is identified as the habitat type with the highest FD after forests and shrublands. Rocky vegetation and screes are the habitat types with the highest endemism in Thermo‐, Meso‐, and Supra‐MVBs, respectively.

The post hoc test evaluating FD across habitat types reveals statistically significant differences between forests/shrublands and screes/steppes. Forests and shrublands exhibit the highest mean FD values, while screes and steppes have the lowest mean FD values, respectively. The relationship between SD and habitat types is not found to be significant according to the ANOVA analysis. However, a significant relationship is observed between endemicity and habitat types. The post hoc test shows that scree habitats are significantly different from forests and shrublands, while steppe habitats are significantly different from all habitat types except screes and dolines. Scree and steppe habitats, characterized by stressful topographic and climatic conditions, have lower mean FD values compared to other habitat types but exhibit higher endemicity rates. Among the vegetation belts, Cryoro‐MVB, which includes these habitats, stands out as the belt with the lowest mean FD values.

### 3.2 Modeling and Dissemination

**FIGURE 6:**
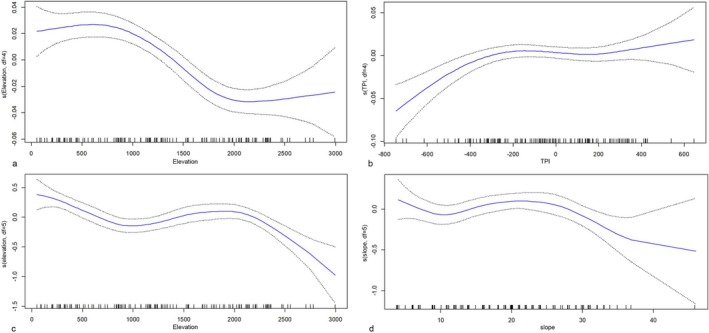
Partial effects plots of GAM showing the influence of elevation (a) and TPI (b) on functional diversity, and elevation (c) and slope (d) on species diversity.

**FIGURE 7:**
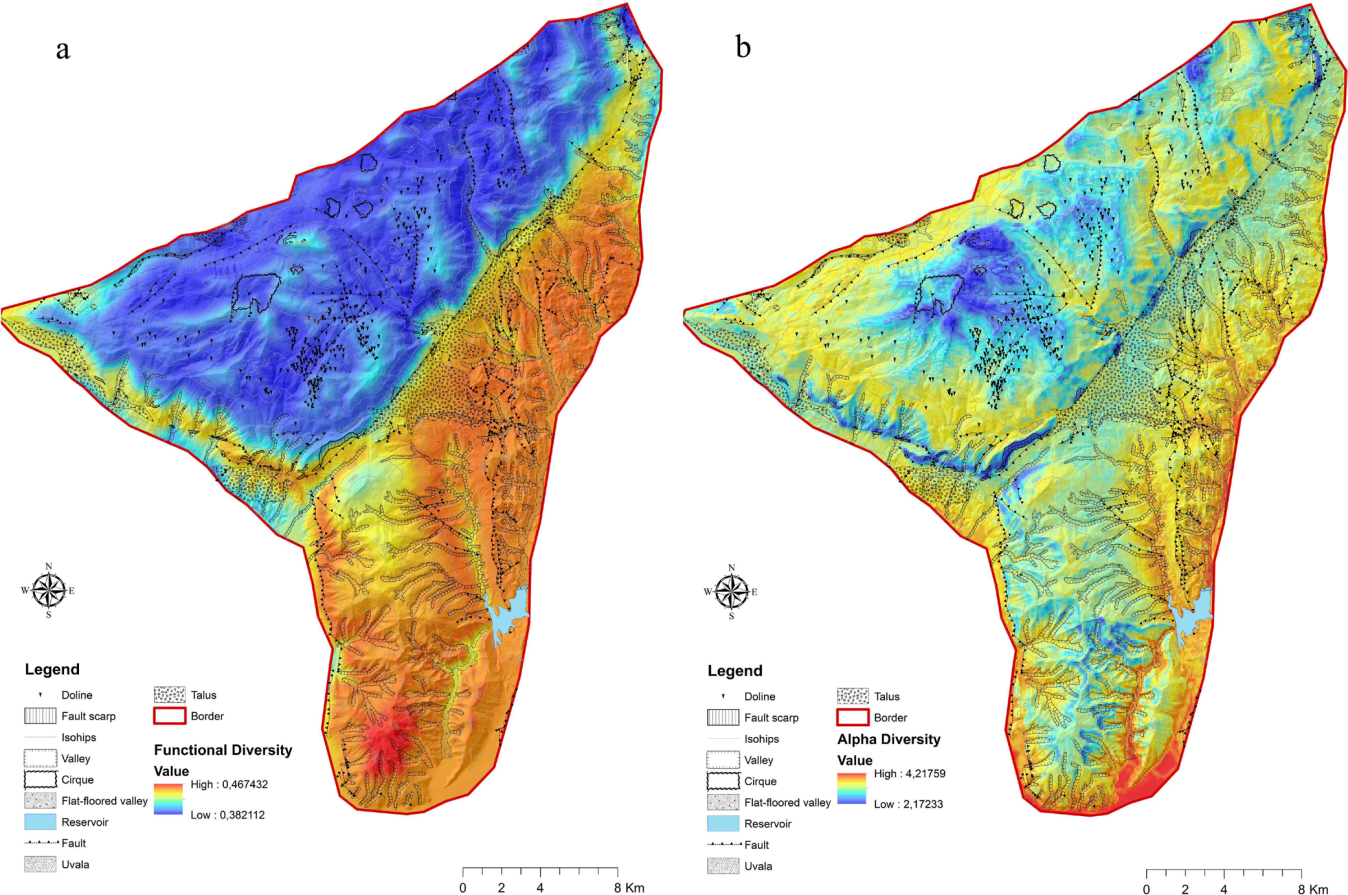
Maps obtained by disseminating the model results of the relationship between functional diversity (a) and species diversity (b) values and environmental variables.

In the GAM analysis, the backward selection method was utilized to derive the model, which was primarily structured by the elevation and TPI variables based on the AIC values. The degrees of freedom for both elevation and TPI were set at 4 in the backward selection method. Table 5 shows the significance test results for the effects of FD and SD in the GAM model. Elevation emerges as a consistently significant predictor for both FD and SD across parametric and nonparametric analyses, with particularly strong effects (*p* < 0.0001 in FD and *p* < 0.00001 in SD for nonparametric effects). TPI significantly influences FD in both parametric (*p* = 0.01765 < 0.05) and nonparametric tests (*p* = 0.00192 < 0.05), highlighting its importance in explaining FD. For SD, slope does not show significance in the parametric test (*p* = 0.1155), but it becomes significant in the nonparametric analysis (*p* = 0.009 < 0.05), suggesting that a nonlinear relationship better captures its effect. Overall, elevation demonstrates the most robust impact across all tests, while TPI and slope show varying levels of significance depending on the diversity metric and the type of test (Table 5). The graphical output from the GAM model and the maps generated from the generalization of the model results can be seen in Figures 6 and 7.

**TABLE 5:** Significance test results of functional diversity and species diversity in the GAM model.

ANOVA Test for parametric effects						
		Degrees of freedom (df)	Sum of squares	Mean squares	*F*	Significance level
FD	s (elevation, df = 4)	1	0.040478	0.040478	52.294	< 0.0001
	s (TPI, df = 4)	1	0.004475	0.004475	5.7807	0.01765
	Residuals	127	0.098305	0.000774		
SD	s (elevation, df = 5)	1	1.7974	1.7974	13.8001	0.0003
	s (slope, df = 5)	1	0.3271	0.3271	2.5119	0.1155
	Residuals	125	16.2798	0.13024		
ANOVA Test for Nonparametric Effects						
FD	s (elevation, df = 4)	3			6.1791	0.00059
	s (TPI, df = 4)	3			5.2403	0.00192
SD	s (elevation, df = 5)	4			8.7616	< 0.00001
	s (slope, df = 5)	4			3.5121	0.009401

In the graphs of the obtained model of the FD index, a partial increasing trend is observed as the elevation rises from approximately 750 to 800 m. Between elevations of 800 and 2000 m, the FD index tends to decrease significantly. Above 2000 m, no marked differentiation in the FD index is evident. The effect of the TPI reveals a positive association between −400 and 200. The confidence intervals for TPI include zero in certain regions, which suggests that the effect may not be statistically significant across the full range. Specifically, in the range between −400 and 200, although the blue line is above zero, the confidence intervals overlap with zero, indicating that the effect might not be significant. Additionally, at very low (−600) or very high (+400) TPI values, the confidence intervals widen significantly and include zero, reflecting lower reliability in the predictions for these ranges. For TPI, the presence of confidence intervals including zero implies that additional tests, such as *p*‐values, are necessary to confirm the statistical significance of its effect in these regions. As seen in Table 5, TPI has a statistically significant effect. For this reason, we categorized TPI ranges into specific intervals and assessed how diversity values vary across these intervals using ANOVA and post hoc tests (Appendix S3). The TPI classification was determined based on the average TPI values of each vegetation belt. The Thermo‐ and Meso‐MVBs, which represent the lowest elevations in the study area, feature a topography shaped by fluvial activity. In general, with increasing elevation in mountainous areas, the average TPI value also increases. The highest TPI values are found in the high mountain belt, which includes summit areas. Among them, the Meso‐MVB has the most negative mean TPI value (−377), followed by Thermo‐MVB (−198), Supra‐MVB (−31), Oro‐MVB (+146), and Cryoro‐MVB (+184). The TPI groups are defined as follows: values of −377 and lower (Group 1); −376 to −198 (Group 2); −197 to −32 (Group 3); −31 to +146 (Group 4); and +147 and higher (Group 5). The TPI values assigned to groups, along with the graphical output from the GAM model (Figure 6), provide a clearer depiction of the trends.

Species diversity initially decreases with elevation up to approximately 1000 m, increases between 1000 and 2000 m, and subsequently declines above 2000 m. SD also rises with slope values between 10° and 20° but declines in steeper areas where slopes exceed 20°. The model highlights that Thermo‐ and Oro‐MVB regions exhibit high SD, whereas alpine sections display lower SD (Figures 5, 6, 7).

## 4 Discussion

The selection of functional traits is fundamental in determining biodiversity patterns, as different traits capture distinct ecological processes. In this study, we focused on five vegetative and three generative functional traits. These traits provide critical insights into how species respond to environmental gradients. For example, in a recent large‐scale study modeling the distribution of Raunkiær's life forms across 546,501 vegetation plots in Europe, Midolo et al. (2024) demonstrated that the proportion of hydrophytes, geophytes, and phanerophytes was predominantly shaped by habitat type, which had a relative influence exceeding 70%, independently of bioclimatic gradients. Clonality, an essential reproductive strategy, enables species to persist through resource sharing and rapid expansion, playing a key role in disturbed or drought‐prone habitats (Dong et al. 2014; Gross et al. 2017). Similarly, nutrient uptake strategies, including mycorrhizal associations and root architecture, determine species' competitive abilities in nutrient‐limited environments (Neumann and George 2010). Other functional traits, such as spinescence, influence herbivore‐plant interactions, reducing browsing pressure and shaping species distributions (Cooper and Ginnett 1998; Bagella et al. 2019). Leaf phenology governs carbon assimilation and water balance, with shifts in phenological patterns serving as key indicators of climate change (Piao et al. 2019). Finally, flowering traits and dispersal strategies regulate reproductive success and species migration, with zoochory and anemochory facilitating habitat colonization under varying ecological conditions (Traveset et al. 2014; Treep et al. 2021). Based on these traits, we interpreted the FD values determined by Rao's Q in relation to variations in SD and endemicity. We grouped our study areas according to vegetation belts and habitat types, evaluating the effects of environmental factors on diversity metrics by integrating our modeling results.

### 4.1 The Complex Relationship Between FD Rao's Q, Species Diversity, and Endemicity (Q1)

The nature of complex ecological communities is assessed by multifaceted influences such as species richness as well as potential changes in the strategies possessed by species within the community and their uniqueness (Kraft et al. 2008). Botta‐Dukát (2005) emphasized that the potential maximum Rao's Q value depends on the number of functionally different species. Additionally, the contribution of rare endemic species to FD shows that these species often have unique functional characteristics and play important roles in ecosystems (Mouillot et al. 2013; Cutts et al. 2023).

The interplay between FD, SD and endemicity highlights the complex ecological dynamics within Mediterranean mountain ecosystems. Endemic species, while contributing uniquely to ecosystem functionality, often co‐occur in habitats with reduced overall FD and SD due to the constraints of their specialized ecological niches (Gorman et al. 2014). The significant negative correlation between endemicity and FD observed in this study suggests that endemic‐rich habitats tend to exhibit lower FD values, likely due to the dominance of specialized species adapted to narrow ecological niches with constrained functional trait combinations. While this pattern aligns with the environmental filtering hypothesis (Keddy 1992), which posits that extreme or isolated conditions select for species with specific adaptations while limiting functional trait diversity, it is also influenced by phylogenetic and biogeographic factors, including speciation, dispersal, and extinction dynamics (Wiens and Donoghue 2004). Additionally, previous studies have demonstrated that endemic‐rich habitats are particularly vulnerable to environmental changes due to their reliance on species with specialized traits and limited dispersal capacities (Pauli and Halloy 2019; Manes et al. 2021).

Similarly, the moderate negative correlation between endemicity and SD reflects the fact that those habitats with high levels of endemism, such as scree and steppe areas, often have lower species richness. These habitats are shaped by strong selective pressures, such as drought resistance and nutrient uptake limitations, which limit the establishment of generalist species while favoring the persistence of specialized endemics (Grime 1977). This supports the notion that endemicity is frequently associated with environments where specialization is prioritized over diversity.

In contrast, forests and shrublands display higher averages for both FD and SD while exhibiting lower endemicity. These habitats likely support generalist species capable of fulfilling broader ecological functions, resulting in greater functional redundancy. This observation aligns with the complementarity hypothesis, which suggests that resource‐rich habitats enhance functional diversity through the complementary traits of co‐occurring species (Loreau and Hector 2001).

Although specific traits of endemic species were not directly examined in the present study, the observed patterns suggest the presence of an endemic syndrome of traits hypothesized by Gorman et al. (2014), characterized by adaptations to harsh or specialized environmental conditions. These syndromes include traits like narrow environmental tolerances and resilience to extreme conditions (Gorman et al. 2014), which, while vital for survival, may limit the range of functional trait combinations within these habitats. This dynamic contributes to the negative correlation between endemicity and FD, as these environments restrict functional trait diversity while fostering unique ecological strategies (Bracken and Low 2012). However, recent evidence challenges this generalization: for example, Behroozian et al. (2020) demonstrated that some rare endemic species can exhibit considerable functional variability across contrasting habitats. Their findings suggest that ecological rarity and endemism are not always associated with narrow functional specialization but may also arise from factors such as recent speciation events and limited dispersal capabilities (Behroozian et al. 2020). While our study indicates that endemic species tend to be functionally adapted to more specialized habitats at a broad scale, further detailed investigations are required to establish comprehensive generalizations on this topic.

Stress factors such as extreme climatic or edaphic conditions limit their capacity to sustain diverse functional groups, making endemic‐rich habitats particularly vulnerable to external disturbances. These findings underscore the ecological and conservation significance of such habitats, as they represent biologically and functionally irreplaceable systems (Pauli et al. 2012; Evangelista et al. 2016). This study's findings are consistent with previous research emphasizing the conservation value of high mountain ecosystems, which are particularly susceptible to climate change and anthropogenic pressures. Despite lower FD and SD, habitats with high endemicity support species with unique functional roles that contribute disproportionately to ecosystem stability and resilience (Ruiz‐Labourdette et al. 2011; Bricca et al. 2021).

### 4.2 Changes in FD Rao's Q and Species Diversity Values With Increasing Elevation (Q2)

One of the most significant distinctions of this study from similar works in the literature is that it includes all plant taxa present in the plots. This approach has enabled us to achieve the most accurate diversity values possible for the studied plots. As indicated in the literature (de Bello et al. 2021), our results show that FD calculated using Rao's Q varies independently of SD. Additionally, the changes in FD calculated in this way can also be interpreted as changes in standardized effect size values along an elevation gradient (de Bello et al. 2021). Consequently, the variations in the FD and SD values we obtained have allowed us to interpret ecosystem dynamics in greater detail. In the GAM graphs we obtained, FD and SD generally show a decreasing trend with increasing elevation, and it is observed that they have almost opposite curves (Figure 6).

The high FD and SD values of the Thermo‐MVB indicate that the environmental conditions of this zone support both species and functional diversity. The low endemism rate suggests that this region provides a suitable habitat for generalized species. Competition and niche differentiation might play a crucial role in shaping the community structure of this zone. For instance, MacArthur and Levins' (1967) niche differentiation theory explains that the high FD in the Thermo‐MVB may be attributed to species utilizing resources in different ways, allowing them to coexist. Additionally, Grime's (1977) stress and competition balance model suggests that in resource‐rich environments like the Thermo‐MVB, competition plays a dominant role in community assembly. Therefore, competition is likely a driving force in the ecological dynamics of this zone.

Despite having lower SD compared to the Thermo‐MVB, the high FD in the Meso‐ and Supra‐MVBs indicates that functional diversity is preserved, suggesting that different mechanisms govern community dynamics in these zones. Such patterns align with Diamond's (1975) limiting similarity theory, which suggests that species in these zones may reduce niche overlap through functional “trait divergence” to minimize direct competition. Additionally, Tilman's (1994) competition and resource use theory highlights how differential resource utilization in such environments can further enhance FD.

Although the Oro‐MVB is rich in SD, its low FD values suggest that habitat filtering mechanisms dominate the ecological dynamics of this zone. Habitat filtering, which leads to species developing similar adaptations to environmental conditions, results in functional convergence (trait convergence) (Weiher and Keddy 1995). Analyses indicate that environmental variables such as the topographic position index (TPI) play a significant role in this filtering process. Körner's (2004) studies on stress factors in mountain ecosystems support the idea that the Oro‐MVB, with its low temperatures and resource limitations, is an ecosystem highly influenced by habitat filtering. This mechanism narrows the range of functional traits in communities, leading to low FD.

The high mountain zones stand out as areas with the lowest FD and the highest endemism rates. This indicates that strong habitat filtering mechanisms are triggered by extreme stress conditions. Körner's (2007) studies on biodiversity in mountainous areas highlight that harsh environmental conditions in these regions allow only species with specific adaptations to survive. Moreover, Grime's (1979) stress tolerance theory suggests that strong stress factors in high mountain zones limit community composition to stress‐tolerant species. The significant decline in SD above 2500 m clearly demonstrates the impact of environmental stress on communities. However, the findings that FD may vary depending on topographic position indicate that topographic complexity supports specific ecological niches even under extreme stress conditions. The sensitivity of these areas, as indicated by maps (Figure 7), underscores the need for conservation efforts in high mountain ecosystems. Myers et al. (2000) emphasize the importance of such areas in their biodiversity hotspot approach, reinforcing their conservation priority.

While our study does not explicitly test different community assembly mechanisms using null models, the observed variation in FD and SD along the elevation gradient provides indirect evidence of their influence. Methods such as SES‐FD null models (Botta‐Dukát and Czúcz 2016), functional diversity metrics (Mouillot et al. 2007), and phylogenetic indices (Webb et al. 2002) could help distinguish between habitat filtering, limiting similarity, and niche differentiation more explicitly. However, given the broad ecological focus of this study, our approach relies on established theoretical frameworks to interpret diversity patterns. Future research could incorporate these additional methodologies to further disentangle assembly processes.

### 4.3 The Effect of Topography on FD Rao's Q in Mountainous Areas (Q3)

According to our GAM results, the most influential environmental variable on FD after elevation is the TPI. Classifying the geomorphological units of the area is critical for understanding the ecological characteristics of the site. Generally, glacial forms, karstic forms, steep slopes, slope debris, narrow and deep incised valleys, and riverbeds constitute the common geomorphological units of the area. The study area can be divided into (i) low mountainous areas (0–1200 m) where river shaping is effective, (ii) characteristic middle mountainous areas (1200–2000 m) formed by tectonism and mass movements with steep slopes and talus, and (iii) high mountainous areas (2000 m and above) where karstic shaping is effective (Figures 6 and 7).

The low areas below 1200 m have a topography characterized by fluvial activity, and the average TPI value is negative. Above approximately 1200 m, the topography rises rapidly where the Beydağları fault passes, and steep slopes are developed in the middle mountainous areas where the slope is high. This area is characterized by limestone slopes where TPI values increase positively, and talus deposits (slope debris) where TPI values generally increase negatively, providing a transitional feature in terms of TPI values. In high mountainous areas, surface runoff is low, and karst dissolution processes are effective. Karstic forms (e.g., dolines) are commonly found up to 2700 m. Due to the less pronounced fluvial activity, the degree of incision is low in this area, and therefore TPI values are generally positive, resulting in a generally convex appearance. The obtained maps (Figure 7) facilitate a better understanding of this complex topography.

Depending on a certain range of TPI values, specific topographic conditions (such as slope, aspect, or elevation) can become more uniform. This uniformity can lead to a decrease in the diversity of other topographic variables within that TPI range, affecting factors such as water drainage, soil types, and microclimatic conditions, which in turn can impact biological diversity and ecosystem processes in these areas (Báez et al. 2022). Báez et al. (2022) highlighted that topography in montane forests plays a crucial role in structuring plant communities around ecotones by amplifying stress gradients and creating mosaics of species with different thermal and precipitation optima, thereby increasing community complexity at the landscape scale. Similarly, our findings emphasize a clear relationship between TPI and FD, highlighting its function as an ecological filter that shapes community assembly. Our findings highlight a clear relationship between TPI and FD, emphasizing its role as an ecological filter. TPI values in the range of −377 to −31, representing moderately negative regions, are associated with the highest FD values. Conversely, highly positive TPI values (> 146) correspond to significantly lower FD. This pattern aligns with the idea that TPI moderates microclimatic and edaphic factors, including water drainage, soil composition, and slope stability, which are critical for supporting diverse functional traits. The ecological filtering effect of TPI is particularly evident in the Oro‐Mediterranean vegetation belt (Oro‐MVB), where an increase in species diversity (SD) coincides with a marked decrease in FD (Figure 6). The positive average TPI value (+142.3) in this vegetation belt highlights the role of TPI in limiting FD through the homogenization of environmental conditions at higher altitudes. The variation of FD in the high mountain zone depending on TPI (e.g., ridges, valleys, or slope gradients) highlights the importance of topographic heterogeneity and aligns with the findings of Jiménez‐Alfaro et al. (2014), which suggest a preference for micro‐valleys with high snow cover among endemic species. Valleys often serve as microrefugia for plant diversity by providing higher moisture and nutrient availability, whereas ridges or steep slopes may impose greater stress. These topographic differences can intensify ecological filtering processes affecting FD, particularly at high altitudes. The interplay between TPI, elevation, and FD provides a framework for exploring trait‐environment relationships in other biodiversity hotspots, particularly in the context of global climate change.

### 4.4 The “Mosaic of Diversity” in Mosaic Habitats (Q4)

The study area represents a mountainous system characterized by mosaic habitats, where the interplay of various vegetation types contributes to unique patterns of biodiversity. The analysis highlights significant differences in FD and endemicity among habitat types, as demonstrated by the post hoc tests. Specifically, scree and steppe habitats show statistically significant differences in FD and endemicity compared to forests and shrublands. These findings underscore the critical ecological roles played by each habitat type within the mosaic.

Forests dominated by *Pinus brutia* between 0 and 1500 m exhibit the highest FD values but the lowest endemicity. These forests, forming mosaics with steep rocky cliffs and scree habitats, transition into shrublands in the Thermo‐, Meso‐, and Supra‐MVBs (Figure 2). Both forests and shrublands exhibit high averages for FD and SD; however, their endemicity remains low. In contrast, scree and steppe habitats, characterized by stressful environmental conditions, show lower FD and SD but stand out with significantly higher levels of endemism. The rocky vegetation, despite its low SD, ranks just below forests and shrublands in terms of FD, indicating its functional contribution to the system. In this elevation range, screes emerge as the habitat with the highest endemicity value, despite their limited FD and SD. These results reveal a “mosaic of diversity” where each habitat type uniquely contributes to the overall biodiversity pattern, emphasizing the ecological significance of mosaic habitats in Mediterranean mountainous regions (Figures 2 and 5).

The Oro‐MVB, dominated by *Cedrus libani* forests, exhibits exceptional SD richness. Transition zones in this vegetation belt include screes, steppes, and rocky areas situated within or at the edges of forests. Species in this mid‐mountain range differ significantly from those in lower vegetation belts, often confined to narrow elevation ranges due to steep slopes (Figure 2). This habitat's unique topographic and climatic conditions foster high endemism rates, despite forests at this elevation having lower FD compared to lower altitudes.

The Cryoro‐MVB, characterized by steppe, scree, rocky, and doline habitats (Figure 2), represents the lowest FD and SD values across Mediterranean vegetation belts but exhibits the highest levels of endemism. These findings further underscore the vulnerability of high‐mountain habitats, where extreme conditions and isolation create a unique ecological dynamic. The post hoc tests reveal significant differences in TPI values among steppe, scree, and rocky habitats, reflecting their distinct spatial and ecological characteristics. High mountain ecosystems are known to be highly vulnerable to climate change and land‐use modifications due to their limited species pools, restricted functional diversity, and fragmented distributions (Engler et al. 2011; Pauli et al. 2012). Their isolation and extreme environmental conditions not only make them critical for understanding ecological processes but also for prioritizing conservation efforts. As Harrison and Noss (2017) suggested, isolated and fragmented habitats often serve as refugia for endemic species, providing a critical buffer against biodiversity loss in the face of global change. Similarly, Nogués‐Bravo et al. (2007) highlighted the importance of mountainous regions as biodiversity hotspots and emphasized the need for targeted conservation actions in these areas.

## 5 Conclusions

This study highlights the critical role of elevation and topographic complexity in shaping functional diversity, species diversity, and endemicity within a mountainous refuge encompassing all Mediterranean vegetation belts. Topographic complexity creates mosaics where different habitat types coexist over short distances, resulting in a mosaic of diversity. While fluvial processes shape the topography in lower elevations, karstic processes become more dominant in higher elevations, making each vegetation belt uniquely distinct. Our modeling results indicate that elevation is the most influential factor affecting diversity metrics, with functional diversity and species diversity curves exhibiting different trends with increasing elevation. Additionally, the topographic position index has been identified as the most significant process influencing functional diversity, particularly in terms of habitat filtering. In this context, mosaic habitats, which foster heterogeneity in functional diversity, species diversity, and endemism, play a crucial role in defining biodiversity hotspots. Our findings reveal that the Meso‐, Thermo‐, and Supra‐Mediterranean belts are characterized by high functional diversity, whereas the Oro‐ and Cryoro‐Mediterranean belts exhibit high levels of endemism. These differences highlight key ecological processes that should be considered in conservation prioritization.

The strong influence of topographic complexity and elevational gradients on species diversity and functional differentiation has also been documented in other regions with Mediterranean climates, as well as in boreal mountain systems. This suggests that the processes identified in our study are not limited to a regional scale but may also be applicable across the Northern Hemisphere and globally.

## Author Contributions

**Candan Aykurt:** conceptualization (lead), data curation (equal), formal analysis (supporting), funding acquisition (lead), methodology (equal), project administration (lead), resources (lead), writing – original draft (equal). **Kürşad Özkan:** conceptualization (equal), data curation (equal), formal analysis (equal), methodology (equal), visualization (equal), writing – original draft (equal). **Mertcan Gülben:** data curation (equal), formal analysis (supporting), visualization (supporting), writing – original draft (equal). **Özdemir Şentürk:** data curation (equal), formal analysis (equal), methodology (equal), visualization (equal), writing – original draft (equal). **Emirhan Berberoğlu:** data curation (equal), visualization (supporting), writing – original draft (supporting). **Semra Türkan:** formal analysis (equal), methodology (equal), visualization (equal), writing – original draft (equal). **Zeynep Öz:** data curation (equal). **Hasan Akgül:** data curation (supporting). **Ramazan Süleyman Göktürk:** data curation (equal). **Sinem Günaydın:** data curation (supporting). **Muhammet Murat Görgöz:** data curation (supporting).

## Conflicts of Interest

The authors declare no conflicts of interest.

## Supporting information


Appendix S1.

Appendix S2.

Appendix S3.

## Data Availability

All data utilized in this study are included in Appendix [Link], [Link], [Link] The distribution of species and their traits is given in Appendix S1, whereas the environmental variable data are provided in Appendix S2.
