# Supplementary material for: Patterns of Functional Diversity, Species Diversity, and Endemicity Driven by Elevation and Topographic Complexity in a Mediterranean Mountain Refuge
Source: Ecol Evol. 2025 May 7;15(5):e71354. doi: 10.1002/ece3.71354 (PMC12058463; doi:10.1002/ece3.71354)
Supplement: Supplementary file 3 — Appendix S3. [file ECE3-15-e71354-s003.docx]

**Patterns of Functional Diversity, Species Diversity, and Endemicity Driven by Elevation and Topographic Complexity in a Mediterranean Mountain Refuge**

**Candan Aykurt^a^, Kürşad Özkan^b^, Mertcan Gülben^c^, Özdemir Şentürk^d^, Emirhan Berberoğlu^e^, Semra Türkan^f^, Zeynep Öz^c^, Ramazan Süleyman Göktürk^a^, Hasan Akgül^a^,** **Sinem Günaydın^c^, Muhammet Murat Görgöz^b^**

^a^Akdeniz University, Faculty of Science, Department of Biology, Antalya, Turkiye, (Corresponding Author), e-mail: candan@akdeniz.edu.tr

^b^Isparta University of Applied Sciences, Faculty of Forestry, Department of Soil Science and Ecology, Isparta, Turkiye

^c^Akdeniz University, Graduate School of Applied and Natural Sciences, Department of Biology, Antalya, Turkiye

^d^Burdur Mehmet Akif Ersoy University, Gölhisar Vocational School, Department of Forestry, Burdur, Turkiye

^e^Akdeniz University, Faculty of Literature, Department of Geography, Antalya, Turkiye

^f^Hacettepe University, Faculty of Science, Department of Statistics, Ankara, Turkiye

**Geospatial Data Processing and Matrix Setup**

In this study, two distinct data matrices were required: one for deriving statistical models during the evaluation phase, and another for disseminating the models, specifically for mapping processes. Initially, coordinate values from field inventory studies were digitized using *ArcGIS 10.8* software. Subsequently, a grid network with dimensions of 100 x 100 meters was created over the study area boundary using the ‘*Create Fishnet*’ tool in *ArcGIS 10.8* to facilitate the mapping of the derived models.

Furthermore, the ‘*Extract Multi Values to Points*’ tool in *ArcGIS* 10.8 was employed to transfer numerical values of all environmental and climatic variables into attribute tables of the point-specific vector layers created. This process established two matrices: the sample area data matrix, which contains numerical values for independent variables used in the statistical analyses, and the dissemination data matrix (grid network layer), which includes numerical values for variables necessary during the model mapping stage. Codes pertaining to topographic variables are presented in Table 1.

**Table 1** Codes pertaining to topographic variables

| **Variable Group** | **Variable Name** | **Code** |
| --- | --- | --- |
| **Topographic Variables** | Elevation | Elevation |
|  | Aspect | Asp |
|  | Slope | Slope |
|  | Topographic Position Index | TPI |
|  | Topographic Wetness Index | twi |
|  | Radiation Index | Radidx |
|  | Solar Illumination Index | Sollidx |
| **Land Classes** | Corine Land Cover | Corine |
| **Bedrock Types** | Limestone | Lime |
|  | Pebble | Pbst |
|  | Chert | Chrt |
|  | Alluvium | Alluv |
|  | Sandstone | Sands |
|  | Volcanic Rock | Volc |
|  | Ophiolite | Ophi |
|  | Basalt | Baslt |
|  | Claystone | Clayst |

**Selection of Climate Variables for Modeling**

In the selection of climate variables for modeling, high correlation among climate variables can lead to multicollinearity issues. Therefore, before proceeding with the modeling processes, it is necessary to identify the variables that have the highest representative capacity. For this reason, Principal Component Analysis (PCA) was applied to 19 bioclimatic variables (Table 2 and Table 3). As shown in Table 3, only three components with an eigenvalue greater than one were found, which together explain a substantial 96.534% of the total variance. When examining the coefficients of the relationship between climate variables and these components, it was found that the variables Bio_1, Bio_5, Bio_6, Bio_8, Bio_9, Bio_10, Bio_11, Bio_14, Bio_17, and Bio_18 have values higher than 0.9, either positive or negative, in component 1 (Table 3).

**Table 2** Variance explained by bioclimatic variables

| **Component** | **Total** | **Variance %** | **Cumulative %** | **Total** | **Variance %** | **Cumulative %** |
| --- | --- | --- | --- | --- | --- | --- |
| **1** | 14.112 | 74.275 | 74.275 | 14.112 | 74.275 | 74.275 |
| **2** | 3.021 | 15.898 | 90.173 | 3.021 | 15.898 | 90.173 |
| **3** | 1.209 | 6.361 | 96.534 | 1.209 | 6.361 | 96.534 |
| **4** | 0.629 | 3.308 | 99.842 |  |  |  |

**Table 3** Component loadings for bioclimatic variables

|  |  | **1** | **2** | **3** |
| --- | --- | --- | --- | --- |
| Bio_1 | Annual Mean Temperature | -0.976 | -0.023 | -0.066 |
| Bio_2 | Mean Diurnal Range | 0.788 | -0.548 | 0.239 |
| Bio_3 | Isothermality | 0.809 | -0.544 | 0.183 |
| Bio_4 | Temperature Seasonality | 0.652 | -0.657 | 0.270 |
| Bio_5 | Max Temperature of Warmest Month | -0.959 | -0.096 | -0.041 |
| Bio_6 | Min Temperature of Coldest Month | -0.980 | 0.058 | -0.098 |
| Bio_7 | Temperature Annual Range (Bio_5-Bio_6) | 0.770 | -0.541 | 0.257 |
| Bio_8 | Mean Temperature of Wettest Quarter | -0.980 | 0.014 | -0.073 |
| Bio_9 | Mean Temperature of Driest Quarter | -0.972 | -0.051 | -0.056 |
| Bio_10 | Mean Temperature of Warmest Quarter | -0.971 | -0.060 | -0.051 |
| Bio_11 | Mean Temperature of Coldest Quarter | -0.980 | 0.017 | -0.076 |
| Bio_12 | Annual precipitation | 0.860 | 0.476 | -0.015 |
| Bio_13 | Precipitation of Wettest Month | 0.653 | 0.709 | 0.170 |
| Bio_14 | Precipitation of Driest Month | 0.900 | -0.032 | -0.434 |
| Bio_15 | Precipitation Seasonality (Coefficient of Variation) | -0.797 | 0.257 | 0.526 |
| Bio_16 | Precipitation of Wettest Quarter | 0.692 | 0.667 | 0.209 |
| Bio_17 | Precipitation of Driest Quarter | 0.900 | 0.037 | -0.440 |
| Bio_18 | Precipitation of Warmest Quarter | 0.911 | -0.010 | -0.401 |
| Bio_19 | Precipitation of Coldest Quarter | 0.688 | 0.662 | 0.233 |

Upon analysis of the coefficients associated with the principal components, it was observed that the variables Bio_1, Bio_5, Bio_6, Bio_8, Bio_9, Bio_10, and Bio_11 exhibited substantial correlations (greater than 0.900) with elevation. Consequently, these variables were excluded from the modeling process to avoid multicollinearity issues, as detailed in Fig. 1. Among the remaining variables, Bio_18 was selected as the representative climate variable for the modeling due to its high component loading and a relatively lower correlation with elevation (less than 0.900).


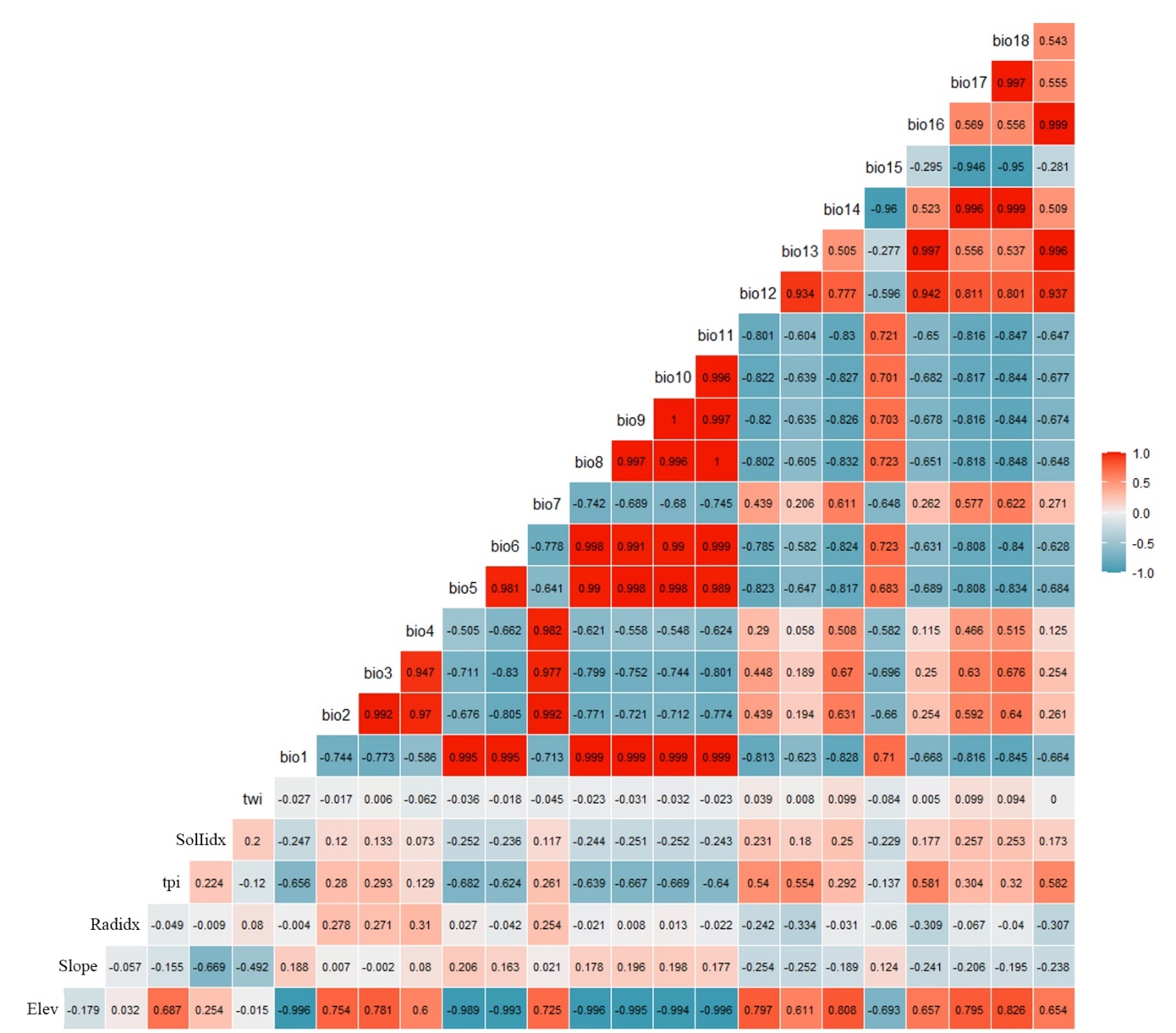


**Fig. 1** Matrix of relationships among independent variables

**Modeling processes**

The comprehensive framework employed for the selection and evaluation of statistical models used to analyze the data is explained in detail below. The data underwent a variable selection process using the stepwise forward selection method, ensuring that only the most predictive variables were included. The dataset was then split into training and testing sets to facilitate robust model training and evaluation, typically utilizing a 10-fold cross-validation approach repeated 100 times to enhance the reliability of the model performance estimates.

Various regression models were trained and evaluated, including Multiple Linear Regression (MLR), Partial Least Squares Regression (PLSR), Ridge Regression (RR), Regression Trees (RT), Multivariate Adaptive Regression Splines (MARS), Generalized Additive Models (GAM), Beta Regression (BR), Quantile Regression (QR), Huber Regression (Huber), and MM-estimators (MM). These models were assessed based on several statistical metrics: the Coefficient of Determination (R²), Root Mean Squared Error (RMSE), Mean Squared Error (MSE), Akaike Information Criterion (AIC), and Corrected Akaike Information Criterion (AICc).

Models were then ranked according to the smallest values of these evaluation metrics to determine the most effective models. The final model, in this case, a Generalized Additive Model (GAM), was chosen based on its superior performance across these criteria, ensuring the model not only fit the data well but also accurately predicted new data, thus optimizing both the validity and practical utility of the model.

**Assessment of Relationships Between Functional and Species diversity and Independent**

**Variables**

Prior to initiating the modeling processes for the functional diversity (FD) components, scatter plots were utilized to examine whether there were linear or curvilinear relationships between FD and the independent variables (Fig. 2 and 3).


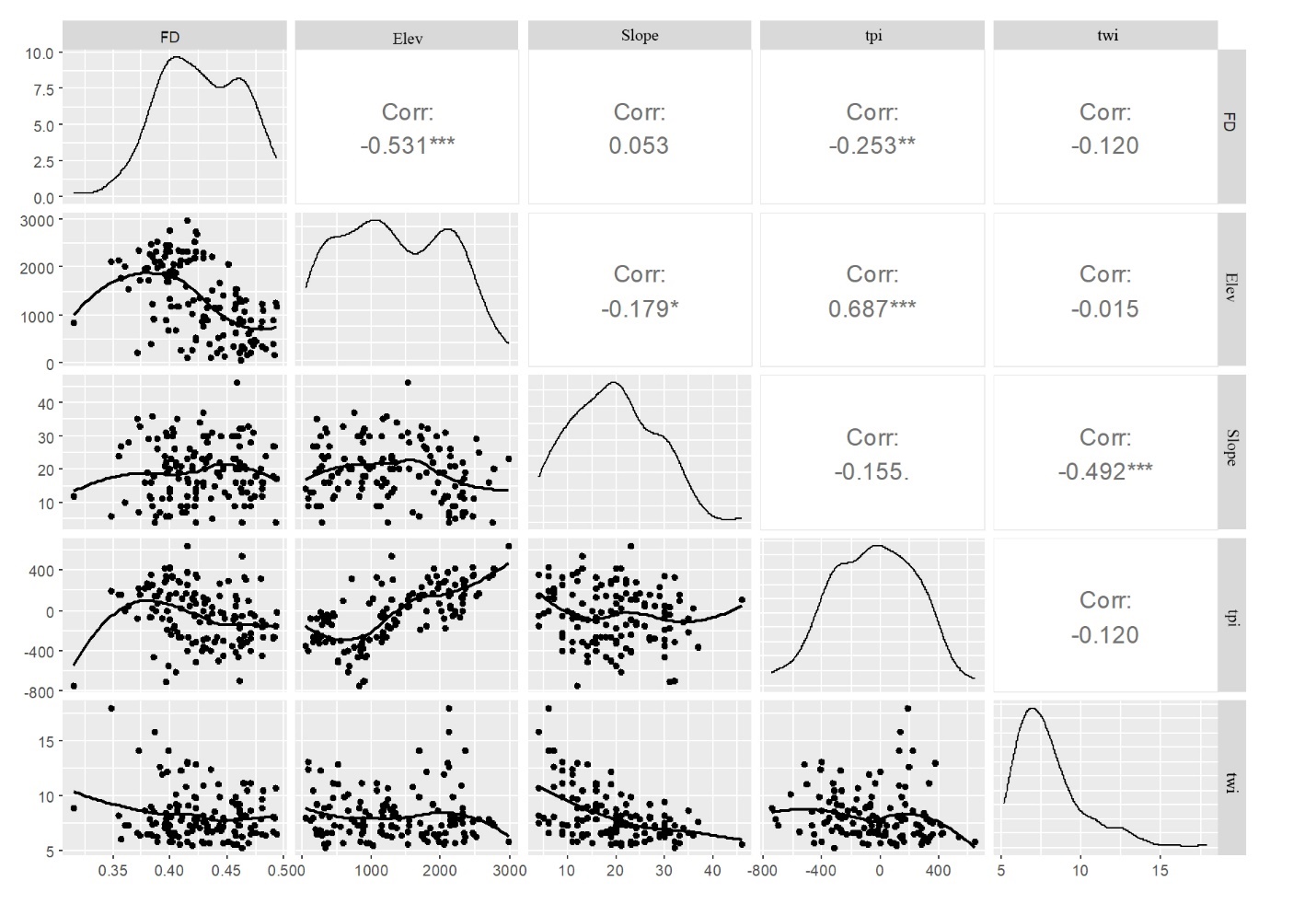


**Fig. 2** Scatter plot of dependent (FD) and independent variables (elevation, slope, topographic position index, and topographic wetness index)


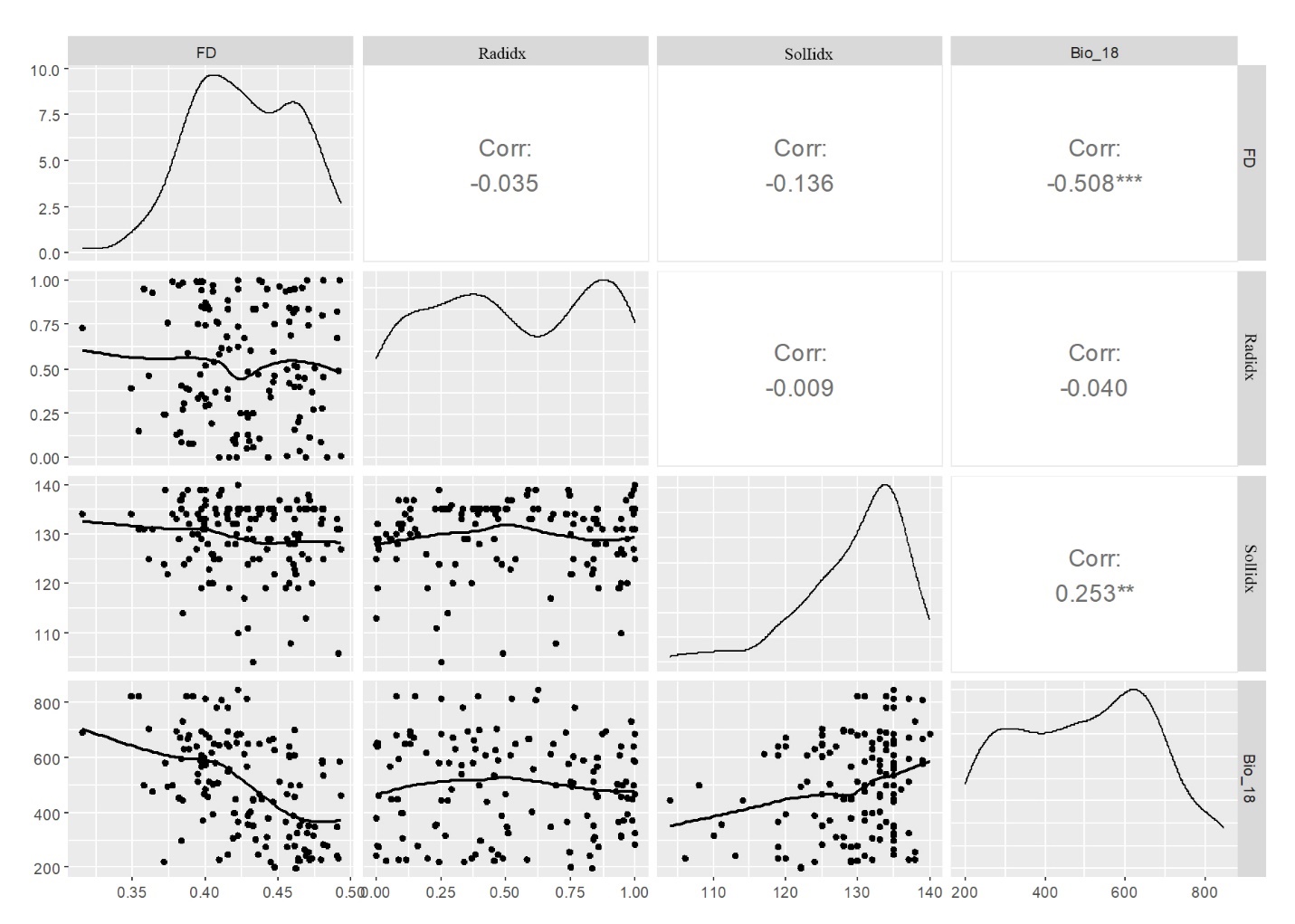


**Fig. 3** Scatter plot of dependent (FD) and independent variables (radiation Index, solar illumination index, and Bio_18).

Fig. 2 and 3 illustrate that there are statistically significant and inverse relationships between FD and the variables elevation, TPI and Bio_18. Furthermore, the relationship between FD and both elevation and TPI is observed to be curvilinear, while the relationship between FD and Bio_18 is linear. The scatter plots strongly indicate that models incorporating curvilinear relationships are likely to achieve greater success in accurately modeling the diversity components of FD.

**Fig. 4** Scatter plot of dependent (SD) and independent variables (elevation, slope, radiation index and topographic position index).

**Fig. 5** Scatter plot of dependent (SD) and independent variables (solar Illumination Index, topographic wetness index, radiation index and topographic position index).

Figures 4 and 5 illustrate the relationships between species diversity and various environmental variables, including elevation, aspect, slope, topographic position index (TPI), topographic wetness index (twi), Radidx, and Sollidx. The correlation coefficients indicate the strength and direction of these relationships.

**Multiple Linear Regression (MLR)**

When the assumptions of the multiple linear regression model were met, a multiple linear regression analysis was conducted to explore the relationship between FD values and environmental factors using the ‘MuMIn’ package in R. The best model was selected based on the smallest Akaike Information Criterion (AIC) values provided by the ‘MuMIn’ package, which also confirmed that all variables included were statistically significant.

The results of the multiple linear regression (MLR) analysis identified elevation and TPI as the variables retained in the model. The contributions of these variables were found to be statistically significant (p < 0.05), and the coefficients along with the constant term for predicting FD are provided in Table 4. According to the t values, the elevation variable contributed most significantly to the model.

**Table 4** MLR model results of FD

| **Variables** | **Coefficients** | **Standard Error** | **t value** | **Significance Level** |
| --- | --- | --- | --- | --- |
| Elevation | -0.00003186 | 0.000004 | -6.816 | <0.00001 |
| TPI | 0.00002723 | 0.000012 | 2.137 | 0.034 |
| Constant | 0.47 | 0.007096 | 66.241 | <0.00001 |

**Partial Least Squares Regression (PLSR)**

The PLSR model analyzing the relationship between FD values and environmental factors was estimated using the ‘pls’ package in R. Upon examining the results, the elevation variable was found to significantly influence the model. Fig. 6 shows that the smallest values for Mean Squared Error (MSE) and Root Mean Squared Error (RMSE) occur when the number of components is between 1 and 6. Selecting a single component, which explained 100% of the variance, was deemed appropriate due to its comprehensive explanatory power. The contribution of all variables included in the model was found to be statistically significant, and the coefficients and constant term for predicting FD are provided in Table 5.


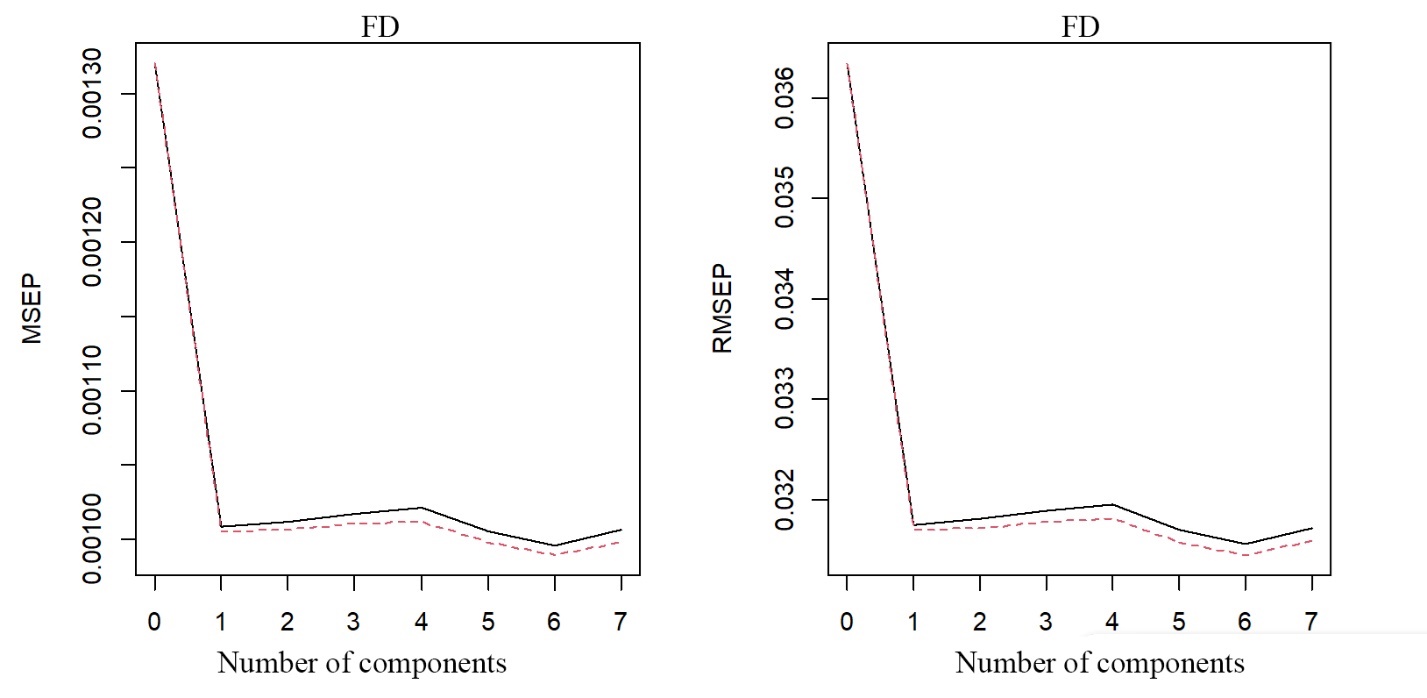


**Fig 6**. Determination of the number of components using cross-validation (CV)

**Table 5** PLSR model results of FD

| **Variables** | **Coefficients** | **Standard Error** | **t value** | **Significance Level** |
| --- | --- | --- | --- | --- |
| Elevation | -0.000025 | 0.000003 | -7.262 | <0.00001 |
| Constant | 0.4597 | 0.005263 | 87.343 | <0.00001 |

**Ridge Regression (RR)**

Ridge regression adjusts the regression estimates by introducing a bias that stabilizes the variance, enhancing the reliability of predictions especially when predictors are highly correlated. The ridge regression analysis between FD values and environmental factors was conducted using the ‘lmridge’ package in R. The ridge trace plot was also generated using the ‘lmridge’ package.

As a result of the ridge regression (RR) analysis, the model was primarily influenced by the variables elevation and Bio_18. The coefficients for these variables, along with the constant, in relation to predicting FD are provided in Table 6. The contributions of the variables included in the model were found to be statistically significant, with the elevation variable making the most substantial contribution according to its t value.

**Table 6** RR model results of FD

| **Variables** | **Coefficients** | **Standard Error** | **t value** | **Significance Level** |
| --- | --- | --- | --- | --- |
| Elevation | -0.1313 | 0.0361 | -3.6366 | 0.0004 |
| Bio_18 | -0.0959 | 0.0361 | -2.6563 | 0.0089 |
| Constant | 221.775 | 51.0166 | 4.3471 | <0.00001 |

In ridge regression analysis, the lambda parameter is chosen as the optimal k-value where the coefficients stabilize in the ridge trace plot. As seen in Fig. 7, since the coefficients begin to stabilize at k=0.2, this value was selected as the optimal k.


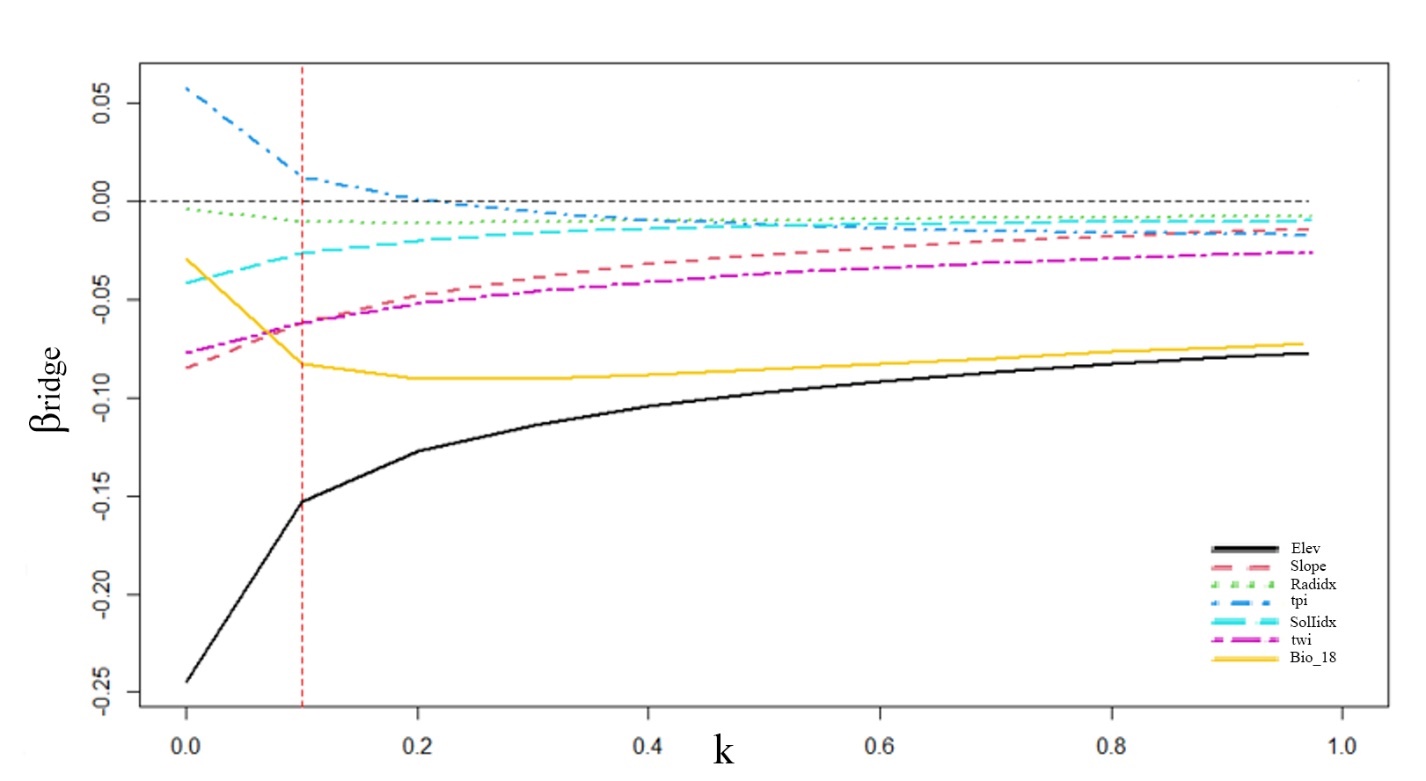


**Fig. 7** Ridge trace plot for variable coefficients across different values of regularization parameter k.

**Regression Tree (RT)**

The regression tree model that investigates the relationship between FD values and environmental factors was developed using the ‘rpart’ package in R. When the ‘minsplit’ parameter is set to 50, the resulting tree model is structured by the variables elevation and Bio_18 (Fig. 8). The elevation variable contributed the most to the model. According to the tree model, the highest diversity values are found in areas where the elevation is less than 1542 meters and Bio_18 is less than 353.6. Conversely, the lowest diversity values are observed in areas with elevations exceeding 1542 meters. The proportions of variance explained by the tree model for both the training and test datasets are shown in Table 14.


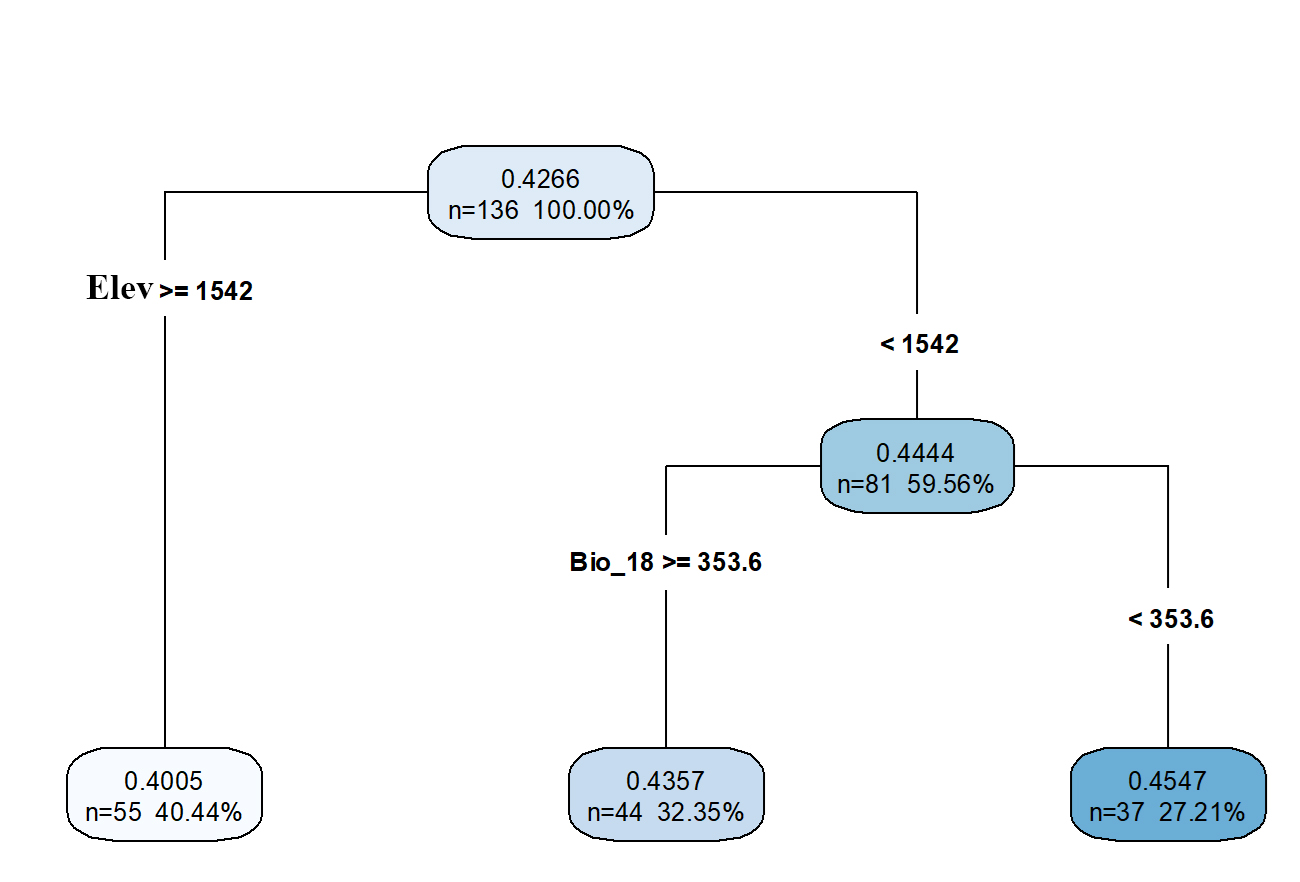


Fig. 8 Regression tree model illustration for FD

**Multivariate Adaptive Regression Splines (MARS)**

The MARS model was employed to analyze the relationship between FD values and environmental variables, utilizing the ‘earth’ package in R. Within this package, the best model was identified using the ‘forward’ selection method (pmethod=forward).

The degree of interaction in the MARS approach was set to 1 based on the Generalized Cross-Validation criterion (GCV) (Table 7). In the MARS method, the forward selection criterion identified elevation, TPI, twi, and Bio_18 as the variables structuring the model. The variables that contributed the most to the model, in order, were elevation, TPI, twi, and Bio_18. The coefficients of the variables entered into the model for predicting FD are presented in Table 8

**Table 7** GCV values associated with model degrees

| **Degree** | **GCV** | **Degree** | **GCV** |
| --- | --- | --- | --- |
| 1 | 0.0008143315 | 6 | 0.0008418765 |
| 2 | 0.0008531048 | 7 | 0.0008418765 |
| 3 | 0.0008583233 | 8 | 0.0008418765 |
| 4 | 0.0008418765 | 9 | 0.0008418765 |
| 5 | 0.0008418765 | 10 | 0.0008418765 |

**Table 8** Results of the MARS model for FD

| **Main Functions** | **Coefficients** | **Standard Error** | **Significance Level** |
| --- | --- | --- | --- |
| max(0, Elevation-880) | -0.00008 | 0.000029 | 0.004480 |
| max(0, Elevation-1903) | -0.00017 | 0.000044 | 0.000094 |
| max(0, 2147-Elevation) | -0.00005 | 0.000021 | 0.009380 |
| max(0, twi-11) | 0.03307 | 0.027250 | 0.227230 |
| max(0, -453.8-TPI) | -0.00024 | 0.000058 | 0.000057 |
| max(0, Elevation-1537) | -0.00008 | 0.000042 | 0.050301 |
| max(0, twi-8.8) | 0.01934 | 0.007654 | 0.012770 |
| max(0, twi-10.4) | -0.06050 | 0.031970 | 0.060560 |
| max(0, 391.9-Bio_18) | 0.00027 | 0.000095 | 0.004290 |

As illustrated in Fig. 9, the FD value shows an increase in the elevation range of 0-1000 meters and a decrease between 1000-2000 meters. It is observed that the FD value increases within the range of -800 to -400 for the TPI variable, and the relationship decreases where the twi variable value is greater than 12 and the Bio_18 value ranges between 200 and 400.


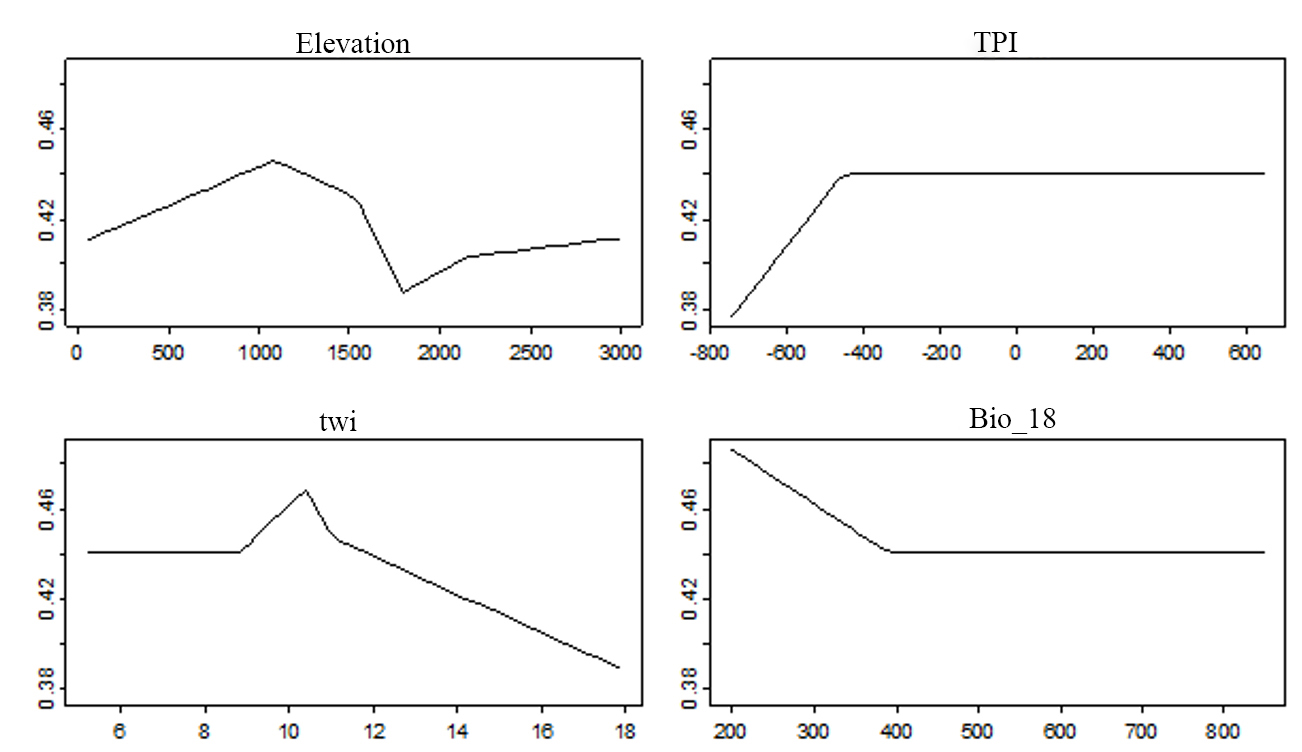


**Fig. 9** MARS model partial effects graph

**Beta Regression (BR)**

The BR analysis determined that the model was primarily driven by the ‘Elev’ variable. The contribution of this variable to the model was found to be statistically significant. The coefficients and constant values of the variable influencing the estimation of FD are presented in Table 10.

**Table 10** BR model results of FD

| **Variables** | **Coefficients** | **Standard Error** | **t value** | **Significance Level** |
| --- | --- | --- | --- | --- |
| Elevation | -0.0001018 | 0.00001405 | -7.249 | <0.00001 |
| Constant | -0.1613 | 0.02137 | -7.550 | <0.00001 |

The values for the explained variance of the model for the training and test datasets in the BR model are presented in Table 10.

**Quantile Regression (QR)**

The quantile regression model examining the relationship between FD values and environmental variables was implemented using the ‘quantreg’ package in R. In the QR analysis, no statistically significant variable was found for the 0.25 quantile; however, statistically significant models were obtained for the 0.50 and 0.75 quantiles. The ‘Elev’ variable was a key determinant in the models for these quantiles. The contribution of this variable to the models was statistically significant, and the coefficients and constant values related to FD estimation are presented in Table 11.

**Table 11** QR model results of FD

| **Quantile=0.50** | | | | |
| --- | --- | --- | --- | --- |
| **Variables** | **Coefficients** | **Standard Error** | **t value** | **Significance Level** |
| Elevation | -0.00003 | 0.00000 | -6.00523 | <0.00001 |
| Constant | 0.46407 | 0.00754 | 61.52326 | <0.00001 |
| **Quantile=0.75** | | | | |
| Elevation | -0.00003 | 0.00000 | -6.32602 | <0.00001 |
| Constant | 0.48476 | 0.00509 | 95.21203 | <0.00001 |

**Huber Regression (Huber) and MM-estimators (MM)**

A key advantage of MM-estimators is that they can be computed more quickly than other robust estimators and are obtained through iterative methods. For investigating the relationship between FD values and environmental factors, robust regression models were fitted using the MASS package in R, employing techniques such as Huber’s M-estimators. Subsequently, model selection and averaging were conducted using the MuMIn package to refine the model inference process.

In the robust regression analysis employing Huber and MM robust estimators, variable selection identified the ‘Elev’ variable as a significant contributor to the models. The statistical significance of the contribution of this variable was established, and the coefficients and constant values for estimating FD based on this variable are detailed in Table 12.

**Table 12** Robust regression model results

| **Huber Estimators** | | | | |
| --- | --- | --- | --- | --- |
| **Variables** | **Coefficients** | **Standard Error** | **t value** | **Significance Level** |
| Elevation | -0.00002604 | 0.00000337 | -7.7185 | <0.00001 |
| Constant | 0.462577 | 0.0052 | 89.6410 | <0.00001 |
| **MM-estimators** | | | | |
| Elevation | -0.00002657 | 0.000003403 | -7.8065 | <0.00001 |
| Constant | 0.463410 | 0.4634 | 89.0441 | <0.00001 |

**Posthoc test Results**

Post hoc test results are presented in Table 13 for FD, SD, and endemiciy across vegetation belts and habitat types. All three diversity metrics were analyzed using the Scheffé post hoc test.

**Table 13** Post hoc test results for FD, SD, and endemicity across vegetation belts and habitat types. Significant differences indicate variations in diversity among specific vegetation belts and habitat types.

| **Groups** | **FD** | | | **SD** | | | **Endemicity** | | |
| --- | --- | --- | --- | --- | --- | --- | --- | --- | --- |
|  | **Vegetation Belt** | | | | | | | | |
|  | **meandiff** | **p_value** | **Significant** | **meandiff** | **p-adj** | **Significant** | **meandiff** | **p_value** | **Significant** |
| **1-2** | 0.0126 | 0.1261 | False | 0.3776 | 0.0009 | **True** | -8.3814 | 0.0004 | **True** |
| **1-3** | 0.0083 | 0.3132 | False | 0.3559 | 0.0017 | **True** | -8.7041 | 0.0002 | **True** |
| **1-4** | 0.0493 | < 0.001 | **True** | 0.1892 | 0.1103 | False | -19.2277 | < 0.001 | **True** |
| **1-5** | 0.0488 | < 0.001 | **True** | 0.3676 | 0.0005 | **True** | -21.6076 | < 0.001 | **True** |
| **2-3** | -0.0043 | 0.6024 | False | -0.0217 | 0.8470 | False | -0.3227 | 0.8895 | False |
| **2-4** | 0.0367 | < 0.001 | **True** | -0.1883 | 0.1149 | False | -10.8463 | < 0.001 | **True** |
| **2-5** | 0.0362 | < 0.001 | **True** | -0.0100 | 0.9236 | False | -13.2262 | < 0.001 | **True** |
| **3-4** | 0.0410 | < 0.001 | **True** | -0.1667 | 0.1626 | False | -10.5236 | < 0.001 | **True** |
| **3-5** | 0.0405 | < 0.001 | **True** | 0.0117 | 0.9108 | False | -12.9035 | < 0.001 | **True** |
| **4-5** | -0.0005 | 0.9518 | False | 0.1783 | 0.1107 | False | -2.3800 | 0.3015 | False |
| **Habitat Type** | | | | | | | | | |
| **1-2** | 0.00631 | 0.48432 | False | 0.09854 | 0.75411 | **True** | -0.40051 | 0.87761 | False |
| **1-3** | 0.00881 | 0.37409 | False | 2.14063 | 0.14590 | False | 4.53965 | 0.11332 | False |
| **1-4** | 0.04361 | 0.01820 | False | 4.08288 | 0.04541 | **True** | 4.82622 | 0.36021 | False |
| **1-5** | 0.02251 | 0.01575 | **True** | 2.83435 | 0.09471 | False | -6.38863 | 0.01743 | **True** |
| **1-6** | 0.05161 | < 0.001 | **True** | 0.80567 | 0.37109 | False | -12.38678 | 0.00004 | **True** |
| **1-7** | 0.05370 | < 0.001 | **True** | 0.25363 | 0.61540 | False | -17.79821 | < 0.001 | **True** |
| **1-8** | 0.04923 | 0.00108 | **True** | 2.52113 | 0.11480 | False | -10.59479 | 0.01383 | False |
| **2-3** | 0.00249 | 0.79509 | False | 1.47046 | 0.22751 | False | 4.94016 | 0.07620 | **True** |
| **2-4** | 0.03730 | 0.04108 | False | 3.54071 | 0.06215 | False | 5.22674 | 0.31774 | False |
| **2-5** | 0.01620 | 0.07066 | False | 4.24671 | 0.04135 | **True** | -5.98812 | 0.02099 | False |
| **2-6** | 0.04529 | 0.00001 | **True** | 1.47127 | 0.22738 | False | -11.98626 | 0.00004 | **True** |
| **2-7** | 0.04739 | < 0.001 | **True** | 0.77217 | 0.38120 | False | -17.39770 | < 0.001 | **True** |
| **2-8** | 0.04292 | 0.00374 | **True** | 3.25237 | 0.07367 | False | -10.19428 | 0.01632 | False |
| **3-4** | 0.03480 | 0.06256 | False | 1.46038 | 0.22910 | False | 0.28657 | 0.95729 | False |
| **3-5** | 0.01371 | 0.16315 | False | 9.38774 | 0.00266 | True | -10.92828 | 0.00017 | **True** |
| **3-6** | 0.04280 | 0.00009 | **True** | 4.92169 | 0.02828 | True | -16.92643 | < 0.001 | **True** |
| **3-7** | 0.04489 | < 0.001 | **True** | 4.32374 | 0.03958 | True | -22.33786 | < 0.001 | **True** |
| **3-8** | 0.04042 | 0.00834 | **True** | 6.28684 | 0.01342 | True | -15.13444 | 0.00069 | **True** |
| **4-5** | -0.02110 | 0.24785 | False | 8.28951 | 0.00468 | True | -11.21486 | 0.03422 | False |
| **4-6** | 0.00799 | 0.66844 | False | 6.06549 | 0.01511 | True | -17.21300 | 0.00170 | **True** |
| **4-7** | 0.01009 | 0.57085 | False | 5.34201 | 0.02242 | True | -22.62443 | 0.00002 | **True** |
| **4-8** | 0.00562 | 0.79426 | False | 7.84087 | 0.00590 | True | -15.42102 | 0.01415 | False |
| **5-6** | 0.02909 | 0.00411 | **True** | 0.42129 | 0.51746 | Flase | -5.99814 | 0.03859 | False |
| **5-7** | 0.03119 | 0.00023 | **True** | 1.88543 | 0.17212 | Flase | -11.40957 | < 0.001 | **True** |
| **5-8** | 0.02672 | 0.07057 | False | 0.28983 | 0.59127 | Flase | -4.20616 | 0.32127 | False |
| **6-7** | 0.00210 | 0.81955 | False | 0.27693 | 0.59963 | Flase | -5.41143 | 0.04270 | **True** |
| **6-8** | -0.00237 | 0.87614 | False | 0.89055 | 0.34711 | Flase | 1.79198 | 0.68338 | False |
| **8-7** | -0.00447 | 0.75223 | False | 1.84127 | 0.17719 | Flase | 7.20341 | 0.07941 | False |

**Model selection**

Cross-validation, particularly k-fold cross-validation, is the most commonly used method for model evaluation and selection in machine learning applications. The term ‘cross-validation’ is sometimes used interchangeably by practitioners and researchers with the hold-out method, where data are split into only training and testing sets. The primary goal of cross-validation is to ensure that every sample in the dataset has a chance to be tested.

In k-fold cross-validation, the dataset is randomly divided into k equal parts, known as ‘folds’. Out of these, a single fold is retained as the test data, and the remaining k-1 folds are used as training data to prevent overfitting. This process is repeated k times, with each of the k folds used once as the test data, and the results from all k iterations are averaged to produce a single estimation. The process of 10-fold cross-validation, which is commonly used, is depicted in Fig. 11.
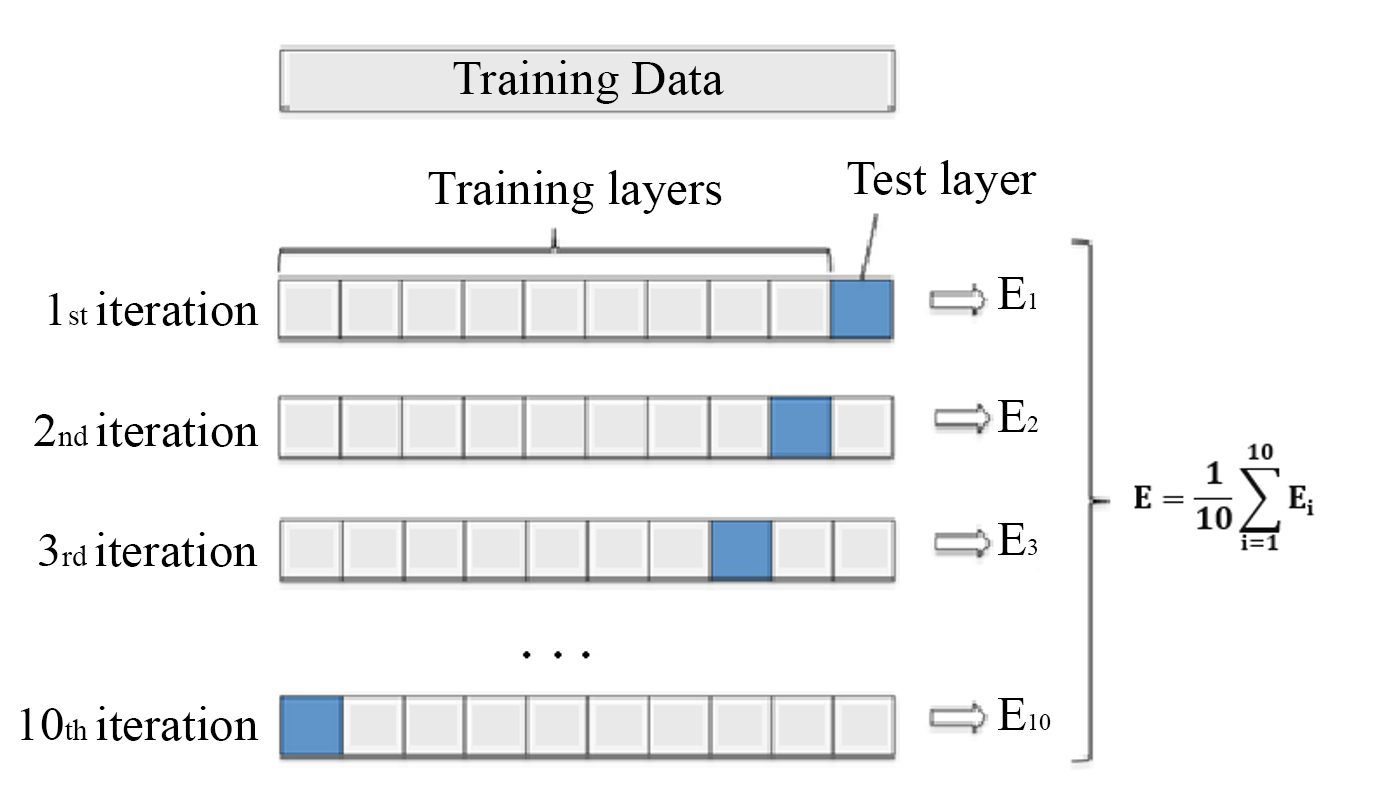


**Fig. 11** Diagram of 10-fold cross-validation process. The E values are used to represent the model performance indicators calculated in each iteration.

The best model relating diversity values to environmental factors was determined using 10-fold cross-validation (CV). In these models, variable selection was conducted using “backward” and “stepwise” methods. From the results of the 10-fold CV, model selection criteria such as R² (formula 1), Mean Absolute Error (MAE) (formula 2), Mean Squared Error (MSE) (formula 3), Root Mean Squared Error (RMSE) (formula 4), Akaike Information Criterion (AIC) (formula 5), and Corrected Akaike Information Criterion (AICc) (formula 6) were calculated. The best models were selected based on the highest R² value and the lowest values for MAE, MSE, RMSE, AIC, and AICc.

| $R^{2}=\left[ \frac{\sum_{i=1}^{n} \left( y_{i}-\bar{y} \right)\left( \hat{y}_{i}-y \right)}{\sqrt{\sum_{i=1}^{n} \left( y_{i}-\bar{y} \right)^{2}\sum_{i=1}^{n} \left( \hat{y}_{i}-\bar{y} \right)^{2}}} \right]^{2}$ | (1) |
| --- | --- |
| $HMO=\frac{\sum_{i=1} \left\vert y_{i}-\hat{y}_{i} \right\vert}{n}$ | (2) |
| $HKO=\sum_{i=1} \left( y_{i}-\hat{y}_{i} \right)^{2}$ | (3) |
| $HKOK=\sqrt{\sum_{i=1}^{n} \frac{\left( y_{i}-\hat{y}i \right)^{2}}{n}}$ | (4) |
| $AIC=n\ln\left( HKO \right)+2p$ | (5) |
| $AICc=n\ln\left( HKO \right)+2p+\frac{2\left( p+1 \right)\left( p+2 \right)}{\left( n-p-2 \right)}$ | (6) |

Here, 𝑌_i_ represents the observed value, Ŷ_i_ denotes the predicted value for the 𝑖^th^ observation as a result of the model, 𝑝 indicates the number of variables, and 𝑛 denotes the number of observations.

When examining the models obtained using different modeling techniques (Table 14), it was determined that the most suitable model according to the evaluation criteria for both the training dataset and the test dataset is the GAM method.

**Table 14.** Results of training and test datasets regarding the models used in modeling FD diversity components.

|  | **R^2^** | | **MAE** | | **MSE** | | **RMSE** | | **AIC** | | **AICC** | |
| --- | --- | --- | --- | --- | --- | --- | --- | --- | --- | --- | --- | --- |
|  | **Training** | **Test** | **Training** | **Test** | **Training** | **Test** | **Training** | **Test** | **Training** | **Test** | **Training** | **Test** |
| **MLR** | 0.306 | 0.267 | 0.024 | 0.024 | 0.001 | 0.001 | 0.030 | 0.031 | -949.329 | -388.412 | -949.239 | -388.317 |
| **PLSR4** | 0.282 | 0.260 | 0.024 | 0.025 | 0.001 | 0.001 | 0.031 | 0.031 | -946.736 | -389.983 | -946.706 | -389.952 |
| **RR** | 0.295 | 0.238 | 0.025 | 0.026 | 0.001 | 0.001 | 0.031 | 0.032 | -941.826 | -386.180 | -941.736 | -386.086 |
| **RT** | 0.456 | 0.276 | 0.020 | 0.024 | 0.001 | 0.001 | 0.028 | 0.031 | -978.497 | -388.125 | -978.191 | -388.031 |
| **MARS** | 0.515 | 0.188 | 0.019 | 0.025 | 0.001 | 0.001 | 0.025 | 0.036 | -982.055 | -364.486 | -980.295 | -363.803 |
| **GAM** | 0.446 | 0.344 | 0.021 | 0.023 | 0.001 | 0.001 | 0.027 | 0.029 | -979.598 | -394.601 | -979.508 | -394.506 |
| **BR** | 0.283 | 0.260 | 0.024 | 0.025 | 0.001 | 0.001 | 0.031 | 0.031 | -946.752 | -389.983 | -946.722 | -389.952 |
| **QR50** | 0.282 | 0.261 | 0.024 | 0.025 | 0.001 | 0.001 | 0.031 | 0.031 | -946.035 | -389.194 | -946.005 | -389.163 |
| **QR75** | 0.282 | 0.263 | 0.031 | 0.031 | 0.002 | 0.002 | 0.039 | 0.040 | -877.031 | -362.847 | -877.001 | -362.815 |
| **HUBER** | 0.282 | 0.262 | 0.024 | 0.025 | 0.001 | 0.001 | 0.031 | 0.031 | -946.325 | -389.893 | -946.295 | -389.862 |
| **MM** | 0.282 | 0.263 | 0.024 | 0.025 | 0.001 | 0.001 | 0.031 | 0.031 | -946.144 | -389.853 | -946.114 | -389.822 |
